# Lactate Metabolic Reprogramming Mediated by CircRNA–LDHA Complex Facilitates Innate Immune Evasion of Liver Cancer

**DOI:** 10.1002/advs.202509989

**Published:** 2025-09-12

**Authors:** Hao Shen, Boqiang Liu, Jing He, Weijun Zhao, Weiqi Li, Lingfeng Ma, Lidan Hou, Yi Wang, Chenqi Jin, Yushun Chang, Jie Lin, Jia Zhao, Binghan Jin, Yuanshi Tian, Xiujun Cai, Liang Shi, Yifan Wang

**Affiliations:** ^1^ Department of General Surgery Sir Run Run Shaw Hospital School of Medicine Zhejiang University Hangzhou 310016 China; ^2^ National Engineering Research Center of Innovation and Application of Minimally Invasive Instruments Hangzhou 310016 China; ^3^ Zhejiang Minimal Invasive Diagnosis and Treatment Technology Research Center of Severe Hepatobiliary Disease Sir Run Run Shaw Hospital School of Medicine Zhejiang University Hangzhou 310016 China; ^4^ Department of Oncology Sir Run Run Shaw Hospital School of Medicine Zhejiang University Hangzhou 310016 China; ^5^ Zhejiang Provincial Key Laboratory of Laparoscopic Technology Zhejiang University Hangzhou 310016 China; ^6^ Department of Endocrinology The Children's Hospital National Clinical Research Center for Child Health School of Medicine Zhejiang University Hangzhou 310053 China; ^7^ Department of Diagnostic Ultrasound & Echocardiography Sir Run Run Shaw Hospital School of Medicine Zhejiang University Hangzhou 310016 China

**Keywords:** circular RNA, immune evasion, lactate metabolism, liver cancer, natural killer cell, protein degradation

## Abstract

Hepatocellular carcinoma (HCC) exhibits a distinctive metabolic profile that engenders a highly immunosuppressive tumor immune microenvironment, posing significant challenges for targeted therapy. Notably, natural killer (NK) cells, which are abundant in the liver and play a crucial role in innate immunity, are attracting growing attention in HCC immunotherapy. Previously, circular RNAs (circRNAs) have emerged as significant regulators in tumor development. Here, an in vitro NK‐cell‐driven tumor evolution model is innovatively established and a novel circRNA, circSMPD4 is identified, which induces lactate metabolic reprogramming in tumor, ultimately promoting immune evasion and metastasis. Lactate dehydrogenase A (LDHA), a main subunit of the critical enzyme in lactate metabolism, is identified as the core effector of circSMPD4's biological function. Mechanistically, circSMPD4 physically combines LDHA to reduce its acetylation levels via SIRT2‐dependent deacetylation and thus preventing its degradation from chaperone‐mediated autophagy‐lysosome pathway. These findings unveil an oncogenic circRNA and elucidate a novel regulatory mechanism by which tumor cell metabolic reprogramming facilitates tumor immune evasion and tumor progression.

## Graphical abstract

CircSMPD4 physically combines with lactate dehydrogenase A (LDHA) and reduces its acetylation level via SIRT2‐dependent deacetylation, thereby inhibiting chaperone‐mediated autophagy‐lysosome degradation of LDHA. Upregulating LDHA leads to increasing lactate production and extracellular export, which in turn suppresses natural killer cell cytotoxicity and induces immune evasion, further promoting hepatocellular carcinoma development.

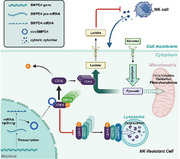

## 1 Introduction

Liver cancer is a global health concern with rising incidence and mortality rates.^[^ ^1^, ^2^, ^3^ ^]^ Hepatocellular carcinoma (HCC) accounts for the majority of primary liver cancers.^[^ ^1^ ^]^ Despite advancements in comprehensive treatment strategies, the prognosis for advanced HCC remains poor.^[^ ^4^ ^]^ Immune evasion is one of the key factors contributing to poor prognosis in HCC. In‐depth research into immune escape mechanisms is crucial for improving treatment and prognosis in HCC.

Immune evasion is considered an inevitable behavior in malignant tumor progression. Solid tumors can attenuate cytotoxic immune cell function by producing a highly immunosuppressive tumor microenvironment (TME).^[^ ^5^ ^]^ Natural killer (NK) cells, the core effector cell of innate immunity,^[^ ^6^, ^7^ ^]^ are enriched in the liver, comprising 25–40% of total intrahepatic lymphocytes.^[^ ^8^ ^]^ This makes NK cells crucial in liver pathophysiology. The quantity, infiltration rate, and functional state of NK cells in the tumor immune microenvironment (TIME) directly affect HCC patient's prognosis.^[^ ^9^ ^]^ Recent studies have shown that interactions between tumor and immune cells in the early stages can directly influence the tumor evolution process and determine tumor fate.^[^ ^10^, ^11^, ^12^ ^]^ Notably, data have revealed that NK cells participate in tumor immunity from early stages of tumor development and play a vital role in tumor progression.^[^ ^13^, ^14^, ^15^ ^]^ However, there remains insufficient in‐depth research on the intrinsic connection between NK cell immune activity and HCC development.

Tumor cells undergo metabolic reprogramming to adapt to the nutrient‐deficient, metabolic waste‐rich TME.^[^ ^14^, ^15^, ^16^ ^]^ A classic example is the Warburg effect, characterized by increased glycolysis and anabolic metabolism with reduced dependence on mitochondrial oxidative metabolism.^[^ ^17^ ^]^ This metabolic pattern forms an immunosuppressive high‐lactate TME. NK cells primarily utilize glucose for metabolism and generating antitumor responses.^[^ ^18^, ^19^, ^20^ ^]^ The high‐lactate TME, which favors tumor cells over NK cells metabolically, creates a vicious cycle promoting tumor growth while suppressing immune responses. Undoubtedly, reshaping tumor cell metabolic patterns and restoring immune cell activity in the TME is crucial for treating solid tumors, including HCC.

l‐lactate dehydrogenase A (LDHA) is a main subunit of LDH, which serves as a critical enzyme in lactate metabolism. Notably, tumor cells prefer to perform Warburg metabolism, characterized by lactate production even in the presence of oxygen.^[^ ^17^ ^]^ Elevated LDHA expression has been observed in various tumor tissues and correlates with poor prognosis in several malignancies.^[^ ^21^, ^22^, ^23^, ^24^, ^25^ ^]^ Current evidence suggests that LDHA level is primarily regulated through transcriptional and posttranscriptional mechanisms,^[^ ^26^, ^27^, ^28^, ^29^ ^]^ with limited research on posttranslational regulation, particularly in HCC.

Circular RNAs (circRNAs), a class of covalently closed single‐stranded RNAs, are characterized by high stability and tissue‐specific expression.^[^ ^30^ ^]^ Mounting evidence suggests their crucial regulatory role in tumor development and progression.^[^ ^31^, ^32^ ^]^ With advancements in technology, circRNA‐based drug platforms have become increasingly mature, and several preclinical trials are currently underway.^[^ ^33^, ^34^, ^35^ ^]^ Meanwhile, our group has conducted in‐depth studies on the functions of circRNA in HCC.^[^ ^36^, ^37^, ^38^ ^]^ In view of the intersection of immunity and metabolism has emerged as a focal point in cancer research,^[^ ^39^, ^40^, ^41^ ^]^ while researches on circRNAs involved in regulating tumor immunity and metabolism, particularly in HCC, remains limited and warrants further investigation. Therefore, we commit to explore how circRNA regulates tumor immunity and metabolic network in HCC.

In this study, we performed a comprehensive analysis of the circRNA transcriptome in human HCC samples and a novel NK‐cell‐induced immune tolerance model. We identified circSMPD4 as an immunosuppressive circRNA in HCC with implications for NK‐cell‐drived tumor evolution. Our findings demonstrate that circSMPD4 alters the metabolic profile of HCC cells, enhancing the Warburg effect and indirectly suppressing NK cell cytotoxicity. Clinically, elevated circSMPD4 expression correlates with poor prognosis in HCC patients. Mechanistically, circSMPD4 physically interacts with LDHA, reducing its acetylation and inhibiting its lysosomal degradation via the chaperone‐mediated autophagy (CMA) pathway. Ultimately, circSMPD4 promotes HCC immune evasion and progression through an LDHA‐mediated tumor metabolism and immunosuppression axis.

## 2 Result

### 2.1 Identification of CircSMPD4 as an Immunosuppressive and Oncogenic CircRNA in NK‐Cell‐Drived HCC Evolution

**Figure 1:**
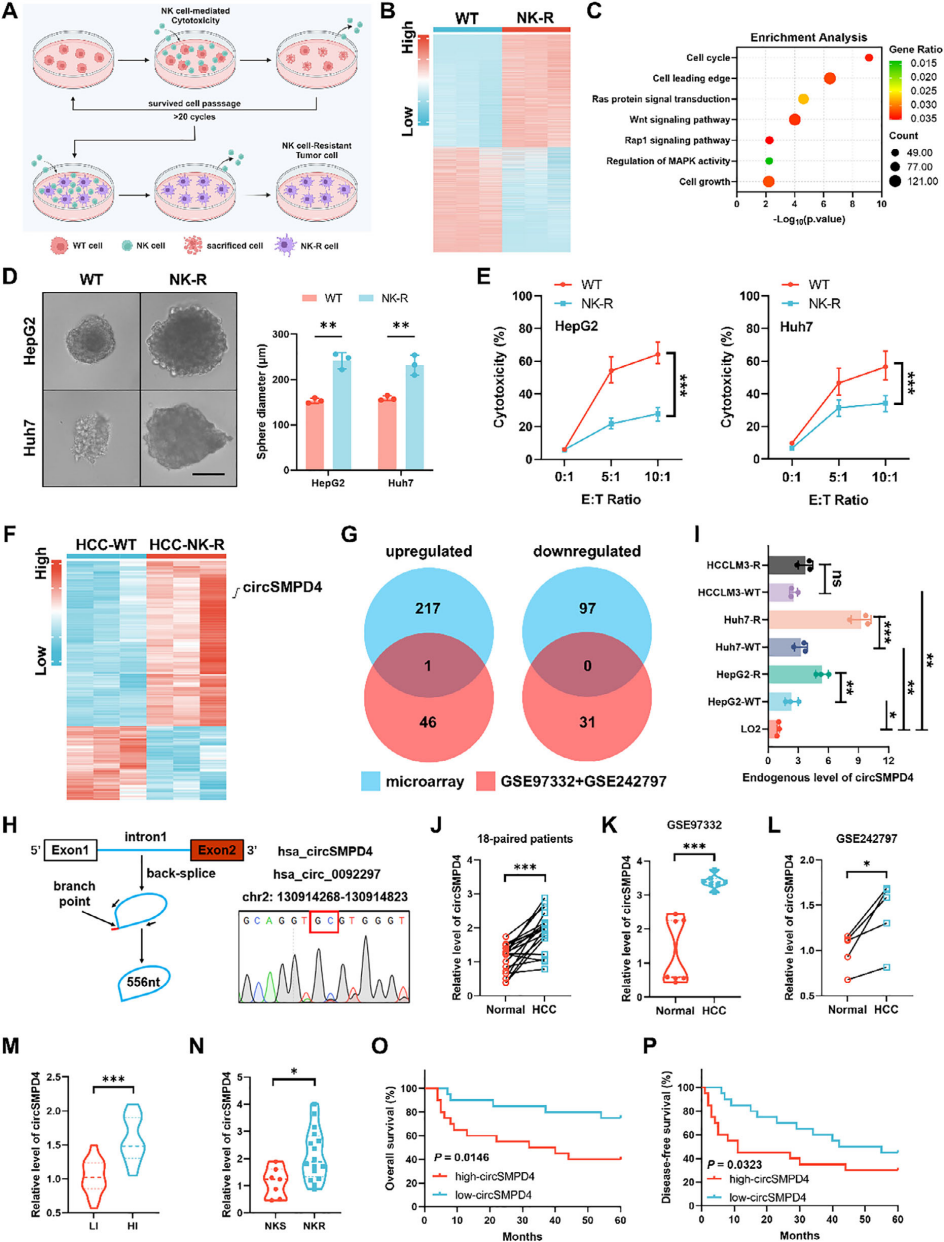
Identification of circSMPD4 as an intronic and immunosuppressive circRNA in NK‐cell‐drived tumor evolution. A) Schematic of the in vitro tumor evolution model: tumor cells were cocultured with NK92MI cells, adjusting the effector‐to‐target (E:T) ratio and incubation time to achieve ≈70% cytotoxicity. Following this, NK cells and dead cells were removed, allowing the surviving tumor cells to grow and undergo passage once. This cycle was repeated over 20 times to generate tumor cells resistant to NK‐cell‐mediated cytotoxicity. This figure was created with BioRender.com. B) A heatmap of differentially expressed genes (*p* < 0.05) between WT and NK‐R cell lines (*n* = 3 independent biological replicates per group). C) Enrichment analysis of transcriptome sequencing on WT and NK‐R cell lines. D) In vitro 3D invasion assays were performed on WT and NK‐R cell lines (*n* = 3 independent biological replicates per group). Representative pictures were shown (left) and the diameter of tumor sphere was measured and analyzed (right, **p < 0.01, unpaired Student's *t*‐test). Scale bar, 100 µm. E) HepG2 and Huh7 cells were subjected to NK cell cytotoxicity assays at various E:T ratios (*n* = 3 independent biological replicates per group), results were quantified using flow cytometry (***p < 0.001, two‐way ANOVA test). F) circRNA microarray analysis of HepG2‐WT cell lines and HepG2‐R cell lines (*n* = 3 independent biological replicates per group). circSMPD4 was denoted by black arrow. G) A Venn diagram showing differentially expressed circRNAs within our microarray data and GSE97332 + GSE242797 dataset. H) The genomic loci and validation of circSMPD4. The fragment containing the back‐spliced junction of circSMPD4 was performed in HepG2‐R cells using divergent primers through quantitative real‐time polymerase chain reaction (RT‐qPCR) and validated by Sanger sequencing. I) The endogenous level of circSMPD4 in several human HCC cell lines (HepG2, Huh7, HCCLM3, and their corresponding NK‐R cell lines, *n* = 3 independent biological replicates per group) and a human liver cell line (LO‐2) (*p < 0.05, **p < 0.01, ***p < 0.001, n.s., not significant, unpaired Student's *t*‐test). J) The expression of circSMPD4 was measured by RT‐qPCR in 18‐paired matched HCC and nontumor tissues (cohort 1, ***p < 0.001, paired Student's *t*‐test). K,L) The expression of circSMPD4 in normal tissues and HCC tissues measured in GSE97332 (K, ***p < 0.001, unpaired Student's *t*‐test) and GSE242797 (L, *p < 0.05, paired Student's *t*‐test) dataset. M,N) RT‐qPCR analysis of circSMPD4 in 32 LI/HI HCC tissues (M, cohort 2, mean ± standard deviation (SD), ***p < 0.001, unpaired Student's *t*‐test), 7 NKS and 16 NKR HCC tissues (N, cohort 3, mean ± SD, *p < 0.05, unpaired Student's *t*‐test). O,P) Kaplan–Meier curves showing overall survival (O) and disease‐free survival (P) of 40 HCC patients (cohort 4) followed up to 60 months. Patients were separated by the median expression level of circSMPD4, using the Gehan–Breslow test.

Emerging investigations have illuminated the pivotal roles of circRNAs as signaling modulators in HCC.^[^ ^32^ ^]^ In our prior work, we constructed in vitro tumor evolution model (refer to the Experimental Section for details) and established NK‐cell‐resistant tumor cells (NK‐R cells), compared to wild‐type tumor cells (WT cells), as a stable tumor cell lines in vitro through long‐term induction by NK92MI cells (**Figure** **1**A). Transcriptome sequencing and subsequent enrichment analysis showed remarkable differences on tumor‐malignancy‐related pathways between WT cells and NK‐R cells (Figure 1B,C), which was also corroborated by in vitro 3D invasion assays (Figure 1D), indicating potential tumor evolution under sustained NK cell immune surveillance pressure. In vitro cytotoxicity assay (refer to the Experimental Section for details) validated that NK‐R cells performed a significant tolerance to NK cell cytotoxicity compared to their WT counterparts (Figure 1E; Figure S1A, Supporting Information). To explore functional circRNAs associated with NK‐cell‐related immune tolerance and tumor progression, we performed circRNA microarray analysis on in vitro tumor evolution model. This analysis revealed 315 differentially expressed circRNAs between the two groups (|log2(fold‐change)| ≥ 0.5, *p* < 0.05), with 218 upregulated and 97 downregulated in NK‐R cell lines (Figure 1F).

We further analyzed publicly available circRNA expression profiles of HCC from the Gene Expression Omnibus (GEO) database (GSE97332 and GSE242797, these two are the most recognized circRNA microarray sequencing databases in HCC research) (Figure S1B,C, Supporting Information). Integration of these datasets identified 78 significantly differentially expressed circRNAs (|log2(fold‐change)| ≥ 1, *p* < 0.05, Figure S1D, Supporting Information). Subsequent comparison with our WT/NK‐R microarray data highlighted circSMPD4 (hsa_circ_0092297) as a candidate of potential functional circRNA (Figure 1G). We validated the circular structure of circSMPD4 using junction‐specific primers and Sanger sequencing (Figure 1H). Consistent with typical circRNA characteristics, circSMPD4 demonstrated enhanced stability compared to its linear counterpart, SMPD4 mRNA (mSMPD4). RNase R digestion assays confirmed circSMPD4's resistance to exonuclease degradation, in contrast to mSMPD4 (Figure S1E, Supporting Information). Additionally, actinomycin‐D‐based RNA stability analysis revealed a significantly prolonged half‐life for circSMPD4 relative to linear mSMPD4 (Figure S1F, Supporting Information), underscoring its unique molecular properties. Notably, circSMPD4 expression exhibited a progressive upregulation trend from normal liver cell to WT tumor cell to highly malignant NK‐R cell, suggesting its potential significance in tumor evolution and immune evasion (Figure 1I).

### 2.2 Elevated CircSMPD4 Expression Correlates with Poor Clinical Outcomes in HCC

To elucidate the clinical relevance of circSMPD4 in HCC, we conducted expression analyses using patient cohort samples from our center. In a cohort of 18 HCC patients (cohort 1), circSMPD4 expression was significantly upregulated in HCC tissues compared to paired normal liver tissues (Figure 1J), corroborating our findings from the GEO datasets (Figure 1K,L). Consistently, we validated with the highly invasive (HI) and low invasive (LI) HCC tissues reported in our previous study^[^ ^36^ ^]^ and found that circSMPD4 is highly expressed in HI HCC tissues compared to LI HCC tissues (cohort 2, Figure 1M). Furthermore, we examined circSMPD4 levels in identified NK‐cell‐sensitive (NKS) and NK‐cell‐resistant (NKR) HCC tissues (cohort 3, refer to the Experimental Section for details). Similarly, circSMPD4 expression was consistently higher in NKR HCC tissues relative to NKS HCC tissues (Figure 1N). Clinical follow‐up data from cohort 4 (Table S1, Supporting Information) revealed that elevated circSMPD4 expression in tumor tissues was significantly associated with reduced overall survival and shortened disease‐free survival (Figure 1O,P).

### 2.3 CircSMPD4 Mediates Resistance to NK Cell Cytotoxicity

To investigate the functional role of circSMPD4 on NK cell cytotoxicity resistance in HCC evolution, we modulated its expression in different cell lines using lentiviral‐mediated overexpression and knockdown strategies. Based on endogenous circSMPD4 levels, we established circSMPD4 knockdown in NK‐R cells (HepG2R, Huh7R) and circSMPD4 overexpression in WT cells (HepG2WT, Huh7WT). Additionally, we constructed circSMPD4 knockout cell lines using CRISPR–Cas9 technology. Successful manipulation of circSMPD4 expression was confirmed by quantitative real‐time polymerase chain reaction (RT‐qPCR) assay (Figure S2A,B, Supporting Information). Importantly, mRNA and protein expression levels of SMPD4 remained unaltered following circSMPD4 modulation, eliminating potential confounding effects in subsequent experiments (Figure S2A–C, Supporting Information).

**Figure 2:**
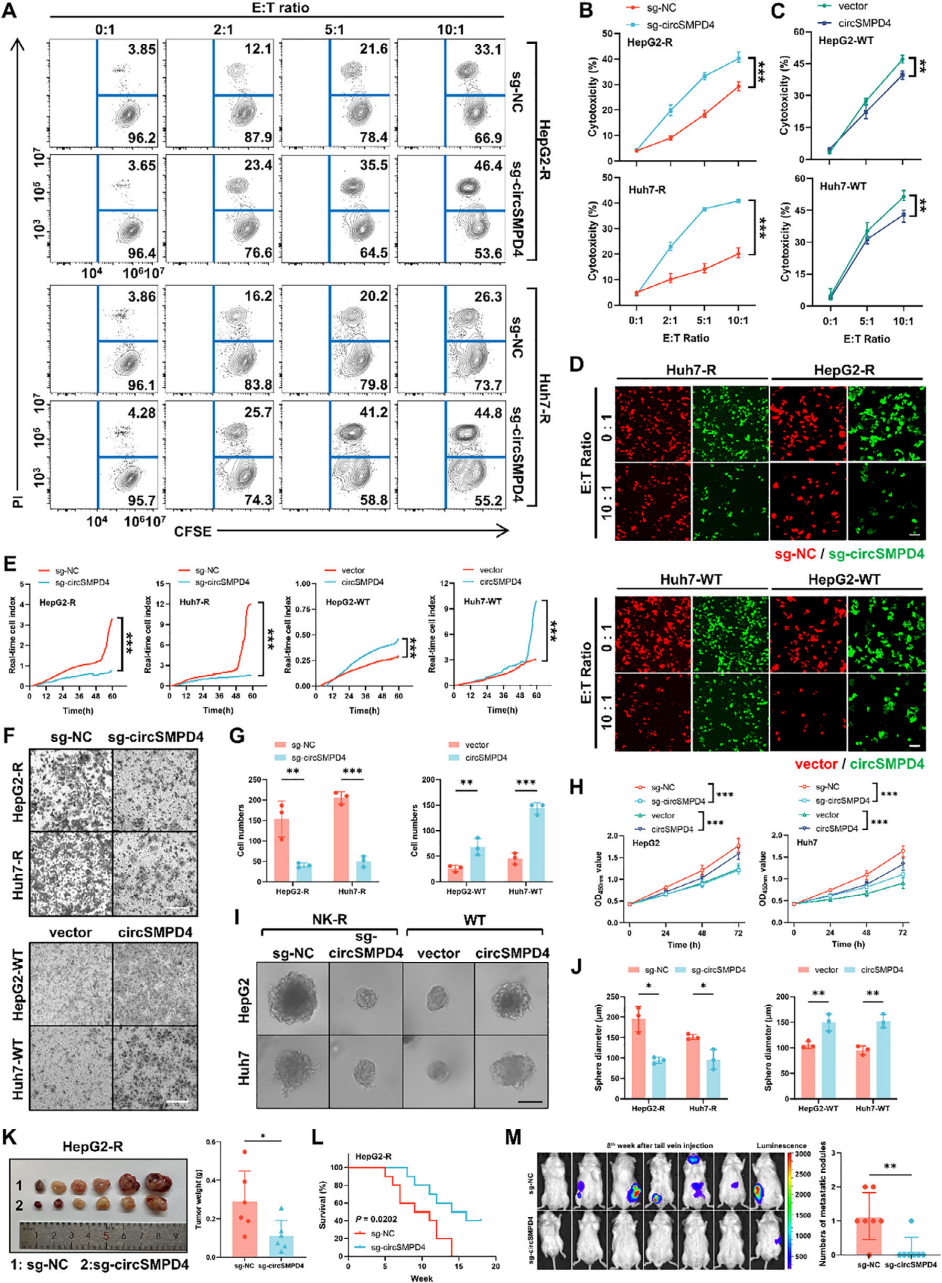
circSMPD4 mediates resistance to NK cell cytotoxicity and enhances tumor malignancy. A) Representative pictures of NK cell cytotoxicity assays on 2 pairs (HepG2 and Huh7) WT and NK‐R cell lines with circSMPD4 knockout using flow cytometry, NK92MI cells were added at different E:T ratios. B,C) 2 pairs (HepG2 and Huh7) WT and NK‐R cell lines with circSMPD4 knockout (B) or overexpression (C) were subjected to NK cell cytotoxicity assays at various E:T ratios, results were quantified using flow cytometry, *n* = 3 independent biological replicates per group (**p < 0.01, ***p < 0.001, two‐way ANOVA test). D) HCC normal control cells were labeled with RFP and circSMPD4 overexpression or knockout cells were labeled with GFP. NK92MI cells were added at different E:T ratios and cocultured for 24 h. After removing NK cells and dead cells, fluorescence images were captured. Representative fluorescence images at different E:T ratios were shown. scale bar, 100 µm. E) Real‐time migration of various tumor cell lines (circSMPD4 knockout or overexpression) was performed on the xCELLigence System and RTCA DP Instrument over 60 h. Data were processed by RTCA software 2.0 (***p < 0.001, two‐way ANOVA test). F,G) Transwell assays with indicated tumor cell lines (*n* = 3 independent biological replicates per group). Representative fields of the porous membranes were shown (F, scale bar, 100 µm), and cell numbers per field were quantified (G, means ± SD, **p < 0.01, ***p < 0.001, unpaired Student's *t*‐test). H) CCK‐8 assays with indicated tumor cell lines (*n* = 3 independent biological replicates per group), OD_450nm_ value were detected every 24 h (***p < 0.001, two‐way ANOVA test). I,J) In vitro 3D invasion assays were performed on indicated tumor cell lines with circSMPD4 knockout or overexpression (*n* = 3 independent biological replicates per group). Representative pictures were shown (I) and the diameter of tumor sphere was measured and analyzed (J, *p < 0.05, **p < 0.01, unpaired Student's *t*‐test). Scale bar, 100 µm. K) Orthotopic tumors of indicated HepG2‐R cells were isolated from mouse liver (left) and the weight of each tumor was quantified (right), *n* = 6 mice per group (mean ± SD. *p < 0.05, unpaired Student's *t*‐test). L) Kaplan–Meier curves showing overall survival of 20 mice after orthotopic tumor transplantation with indicated HepG2‐R tumors followed up to 18 weeks, *n* = 10 mice per group (*p < 0.05, Gehan–Breslow test). M) Left: IVIS images of HepG2‐R tumors at the 8th week after rapid tail vein injection (*n* = 7 mice per group). Right: tumor foci were quantified (mean ± SD, **p < 0.01, unpaired Student's *t‐*test).

To assess the functional consequences of circSMPD4 modulation, we performed in vitro NK cell cytotoxicity assays using cocultures of NK cells and circSMPD4‐manipulated tumor cells. Flow cytometry analysis revealed that while circSMPD4 manipulation did not significantly affect basal cell apoptosis (Figure S2D, Supporting Information), circSMPD4 silencing markedly enhanced the susceptibility of tumor cells to NK‐cell‐mediated cytotoxicity and vice versa (**Figure** **2**A–C; Figure S2E, Supporting Information). These findings were further corroborated by fluorescence‐based NK cell cytotoxicity assays (Figure 2D; Figure S2F, Supporting Information).

### 2.4 CircSMPD4 Enhances Tumor‐Malignancy‐Related Biological Behavior in HCC

Given the significant upregulation of circSMPD4 in HCC tissues compared to adjacent normal liver tissues, we conducted comprehensive in vitro and in vivo experiments to elucidate its functional impact on HCC biological behaviors. The promigratory effect of circSMPD4 was evidenced by dynamic tumor cell migration curves obtained using the xCELLigence Real‐Time Cell Analysis (RTCA) system (Figure 2E) and corroborated by Transwell assays (Figure 2F,G). In vitro cell proliferation assays, including cell counting kit‐8 (CCK‐8) assay (Figure 2H) demonstrated that circSMPD4 significantly promoted HCC growth. Furthermore, 3D invasion assays utilizing Matrigel matrix revealed a remarkable enhancement of tumor cell invasiveness mediated by circSMPD4 (Figure 2I,J).

Due to the lack of conservation of the intronic sequences corresponding to the human circSMPD4 gene in the mouse genome, we were unable to use a conditional knockout mouse model with circSMPD4 knockout in hepatocytes or hepatocellular carcinoma cells for in vivo experiments. Thus, we employed two canonical mouse models to validate the above findings in vivo: orthotopic xenograft mouse model and rapid tail vein injection model. Sustained and effective silencing of circSMPD4 in the injected HCC cells and resultant orthotopic tumors was confirmed by RT‐qPCR assay (Figure S2G,H, Supporting Information). To eliminate confounding effects of liver‐resident NK cells, we utilized immunodeficient NOD/ShiLtJGpt‐Prkdcem26Cd52Il2rgem26Cd22/Gpt (NCG) mice for subsequent experiments. In the 6th week postorthotopic transplantation, tumors derived from circSMPD4‐knockout HCC cells exhibited significantly reduced volume and mass compared to controls (Figure 2K; Figure S2I, Supporting Information). At 18th week postoperation, the circSMPD4‐knockout group demonstrated significantly prolonged overall survival compared to controls (Figure 2L; Figure S2J, Supporting Information). For the metastasis model, circSMPD4‐knockout or control HepG2R cells (1.5 × 10^6^ cells per mouse) were injected into the lateral tail vein of the mouse (*n* = 7 per group). Whole‐body tumor metastasis was evaluated at the 8th week postinjection using an In Vivo Imaging System (IVIS). Strikingly, only 1 out of 7 mice (14.3%) in the circSMPD4‐knockout group developed lung or bone metastases, whereas 6 out of 7 control mice (85.7%) exhibited lung or bone metastases (Figure 2M).

The results of these in vitro and in vivo experiments demonstrate that circSMPD4 can promote HCC progression even in the absence of innate immune pressure. Taken together, our data indicated that circSMPD4 exerts two independent biological functions: facilitating tumor immune evasion and promoting tumor progression.

### 2.5 CircSMPD4 Indirectly Impairs NK Cell Function by Enhancing the Warburg Effect

Subsequently, we sought to explore the precise mechanisms by which circSMPD4 mediates immune tolerance of HCC cells against NK‐cell‐mediated cytotoxicity. Prior studies have established that NK cell cytotoxicity against target cells is largely determined by the balance of NK‐cell‐related activating and inhibitory ligands on the target cell surface.^[^ ^13^ ^]^ Initial analysis of NK cell activating ligand expression in various HCC cell lines revealed that circSMPD4 modulation did not significantly alter their levels (Figure S3A,B, Supporting Information).

**Figure 3:**
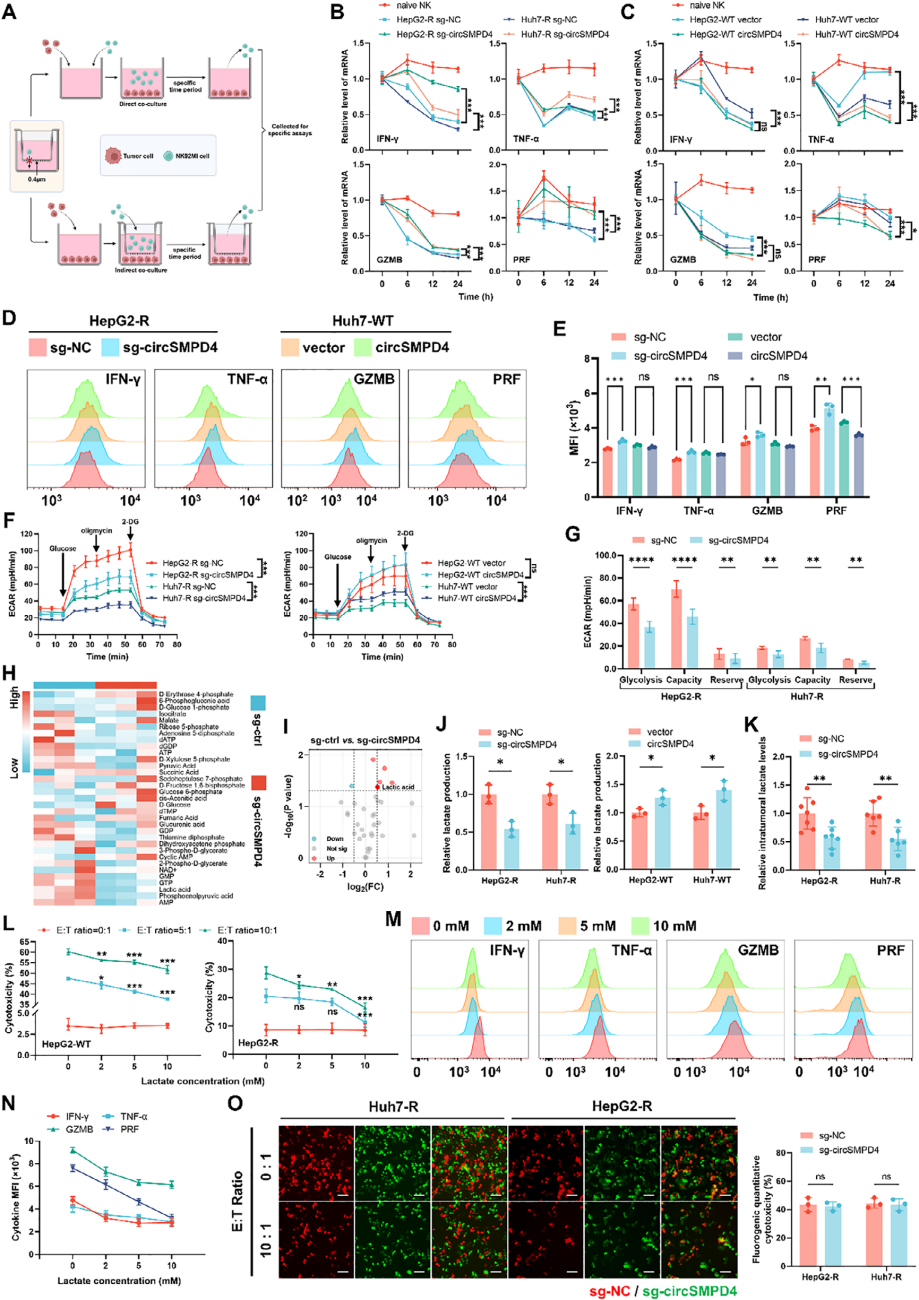
CircSMPD4 impairs NK cell function by enhancing the Warburg effect of HCC. A) Schematic diagram of direct and indirect coculture experiments between NK cells and tumor cells: direct coculture (left): NK cells and HCC cells are plated together at a 1:1 ratio in a single culture dish. Indirect coculture (right): the 0.4 µm chamber (which prevents cell penetration through the membrane) was used to separate the two cell types, with NK cells plated in the chamber and tumor cells plated at a 1:1 ratio in the culture dish. NK cells are collected at specific time points for subsequent experiments. This figure was created with BioRender.com. B,C) Indirect coculture experiments between NK cells and tumor cells with circSMPD4 knockout (B) or overexpression (C). NK92MI cells were collected at specific time points and the expression levels of activating effector molecules were assessed using RT‐qPCR, *n* = 3 independent biological replicates per group (means ± SD, *p < 0.05, ***p < 0.001, n.s., not significant, two‐way ANOVA test). D,E) Plate equal amounts of tumor cells with circSMPD4 knockout or overexpression in culture dishes of the same specifications, and after a specific time, the corresponding supernatants were collected from each cell line for NK cell culture. After culturing for a designated period, NK cells were collected to identify the expression levels of their effector molecules using flow cytometry. Representative pictures were shown (D) and fluorescence intensity was quantified, *n* = 3 independent biological replicates per group (E, *p < 0.05, **p < 0.01, ***p < 0.001, n.s., not significant, unpaired Student's *t*‐test). F) Extracellular acidification rate of tumor cells with circSMPD4 knockout or overexpression, *n* =  4 independent samples per group (mean ± SD, ***p < 0.001, n.s., not significant, two‐way ANOVA test). G) Glycolysis, glycolytic capacity, and glycolytic reserve levels were quantified from glycolysis stress test assays of tumor cells with circSMPD4 knockout, *n* =  4 independent samples per group (mean ± SD, **p < 0.01, ****p < 0.0001, unpaired Student's *t*‐test). H) Targeted metabolomics analysis on central carbon metabolism (CCM) of 3 HepG2‐R sg‐ctrl cells and 3 HepG2‐R sg‐circSMPD4 cells, various metabolites are labeled beside the heatmap. I) A volcano plot of differential metabolites after circSMPD4 knockout. Lactic acid was denoted. J) Measurement of supernatant levels of lactate in tumor cell lines with circSMPD4 knockout or overexpression, *n* =  3 independent samples per group (mean ± SD, *p < 0.05, unpaired Student's *t*‐test). K) Measurement of intratumoral levels of lactate in indicated HepG2‐R or Huh7‐R tumors, *n* = 7 or 6 mice per group (mean ± SD, **p < 0.01, unpaired Student's *t*‐test). L) HepG2‐WT and HepG2‐R cells were subjected to NK cell cytotoxicity assays treated with indicated lactate concentration at various E:T ratios using flow cytometry, cytotoxicity was quantified to perform in the line chart, *n* = 3 independent biological replicates per group (means ± SD, *p < 0.05, **p < 0.01, *** *p* < 0.001, n.s., not significant, two‐way ANOVA test). M,N) The activate levels of effector molecules in NK92MI cells treated with indicated lactate concentration were assessed using flow cytometry. Representative pictures were shown (M), fluorescence intensity was quantified to perform in a line chart (N). O) Tumor normal control cells labeled with RFP and circSMPD4 knockout cells labeled with GFP were mixed in a 1:1 ratio and plated. NK92MI cells were added in different E:T ratios and cocultured for 24 h. After removing NK cells and dead cells, fluorescence images were captured. Left: representative fluorescence images were shown. Scale bar, 100 µm. Right: quantification of NK cell cytotoxicity, *n* = 3 independent biological replicates per group (n.s., not significant, unpaired Student's *t*‐test).

Then, we designed direct and indirect coculture assays to explore how circSMPD4 functioned (**Figure** **3**A). According to numerous authoritative studies, the levels of interferon‐γ (IFN‐γ), TNF‐α, granzyme B (GZMB), and perforin (PRF) can reflect the functional status of NK cells.^[^ ^42^, ^43^, ^44^ ^]^ Therefore, in our subsequent research, the levels of these small molecules will be utilized as biomarkers to evaluate the functional status of NK cells. Intriguingly, coculture assays with NK cells and tumor cells demonstrated a corresponding NK cell functional impairment after altering circSMPD4 expression, irrespective of whether they have a direct physical contact (Figure 3B,C; Figure S3C,D, Supporting Information). Consequently, we postulated that circSMPD4 might indirectly suppress NK cell function through an as‐yet‐unidentified mechanism. To test this hypothesis, we incubated NK cells in conditioned media from tumor cell cultures for a defined period and subsequently assessed NK cell function. Interestingly, we observed results comparable to those obtained in coculture experiments (Figure 3D,E). These observations strongly suggest that circSMPD4 may modulate NK cell function through the secretion or extrusion of a specific factor.

Subsequently, we reanalyzed the transcriptome sequencing data from WT and NK‐R cells and discovered that, in addition to the tumor‐malignancy‐related cell growth and proliferation pathways, the differentially expressed genes were also enriched in cellular energy metabolism pathways (Figure S3E, Supporting Information). Interestingly, during routine cell culture, we observed that circSMPD4 knockout tumor cell lines exhibited reduced acidification of culture media compared to controls, as indicated by phenol red color changes (Figure S3F, Supporting Information). Extensive research experience has demonstrated that changes in cell culture media color often correspond to alterations in cellular metabolism, particularly glycometabolism. Further mitochondrial stress tests and glycolysis stress tests revealed that circSMPD4 significantly impacted overall extracellular acidification rate (ECAR) in tumor cells, rather than overall oxygen consumption rate (OCR) (Figure 3F; Figure S3G, Supporting Information). Notably, the most pronounced effects were observed in glycolysis‐derived extracellular acidification and nonmitochondrial oxygen consumption (Figure 3G; Figure S3H,I, Supporting Information). Integrating our previous research findings, we hypothesized that circSMPD4 might promote metabolic reprogramming in tumor cells.

To elucidate the mechanism by which circSMPD4 modulates glucose metabolism in tumor cells, we conducted a targeted metabolomic analysis of glucose‐metabolism‐related products using our previously established cell lines (Figure 3H). This analysis revealed significant downregulation of 5 metabolites and upregulation of 1 metabolite in circSMPD4 knockout cell lines (Figure 3I). Notably, lactate, a crucial product of lactate metabolism which has been extensively studied and shown to have a significant association with tumor immunity, was among the downregulated metabolites. Several prominent studies suggest that lactate exerts immunosuppressive effects, and its accumulation in the tumor immune microenvironment significantly weakens the activity of cytotoxic immune cells, such as NK cells and T cells, thereby promoting tumor progression.^[^ ^45^, ^46^, ^47^ ^]^ Moreover, given that tumor cells often exhibit a preference for glycolysis over oxidative phosphorylation, resulting in lactate accumulation, we suggested that circSMPD4 might promote the Warburg effect in HCC. Consistent with our metabolomics findings, lactate levels in culture supernatants were reduced in circSMPD4 knockout tumor cell lines and elevated in circSMPD4 overexpression lines (Figure 3J). However, pyruvate levels remained unchanged across circSMPD4‐modulated cell lines (Figure S3J, Supporting Information). Analysis of in vivo orthotopic liver tumors in NCG mice further demonstrated significantly lower intratumoral lactate content in circSMPD4 knockout tumors and accumulation of lactate in circSMPD4 overexpression tumors (Figure 3K; Figure S3K, Supporting Information).

The immunosuppressive character of the tumor microenvironment, partly mediated by metabolite‐induced immune cell dysfunction, is well‐documented.^[^ ^5^, ^48^ ^]^ Recent studies have implicated lactate accumulation in immune cell suppression.^[^ ^14^, ^15^, ^16^ ^]^ To validate this in our model, we performed NK cell cytotoxicity assays under varying lactate concentrations. Our results demonstrated a dose‐dependent decrease in NK cell cytotoxicity against both WT and NK‐R cell lines as environmental lactate levels increased (Figure 3L). Additionally, we found that the elevation of environmental lactate concentration primarily impairs NK cell function by reducing the secretion of cytokines and cytotoxins as well as downregulating activating receptors on NK cells, without significantly affecting NK cell proliferation or the expression of inhibitory receptors (Figure 3M,N; Figure S3L,M, Supporting Information). To establish a causal link between circSMPD4‐induced metabolic reprogramming and NK cell suppression, we employed the glycolysis inhibitor 2‐deoxy‐D‐glucose (2‐DG). Treatment with 2‐DG abrogated the lactate production difference caused by circSMPD4 overexpression (Figure S3N, Supporting Information) and, more importantly, eliminated the NK‐cell‐resistant phenotype induced by circSMPD4 (Figure S3O, Supporting Information). Conversely, exogenous lactate supplementation rescued the increased susceptibility to NK cell cytotoxicity observed in circSMPD4 knockout cells (Figure S3P, Supporting Information). Intriguingly, coculture of control and circSMPD4‐manipulated cell lines with NK cells in a shared environment eliminated previously observed differences in cytotoxicity (Figure 3O; Figure S3Q, Supporting Information).

Collectively, these data provide compelling evidence that circSMPD4 suppresses NK cell function by promoting lactate metabolism reprogramming in HCC, unveiling a novel mechanism by which circRNA‐induced metabolic alterations modulate innate immune responses.

### 2.6 LDHA: A Key Interactor Functionally Relevant to CircSMPD4

**Figure 4:**
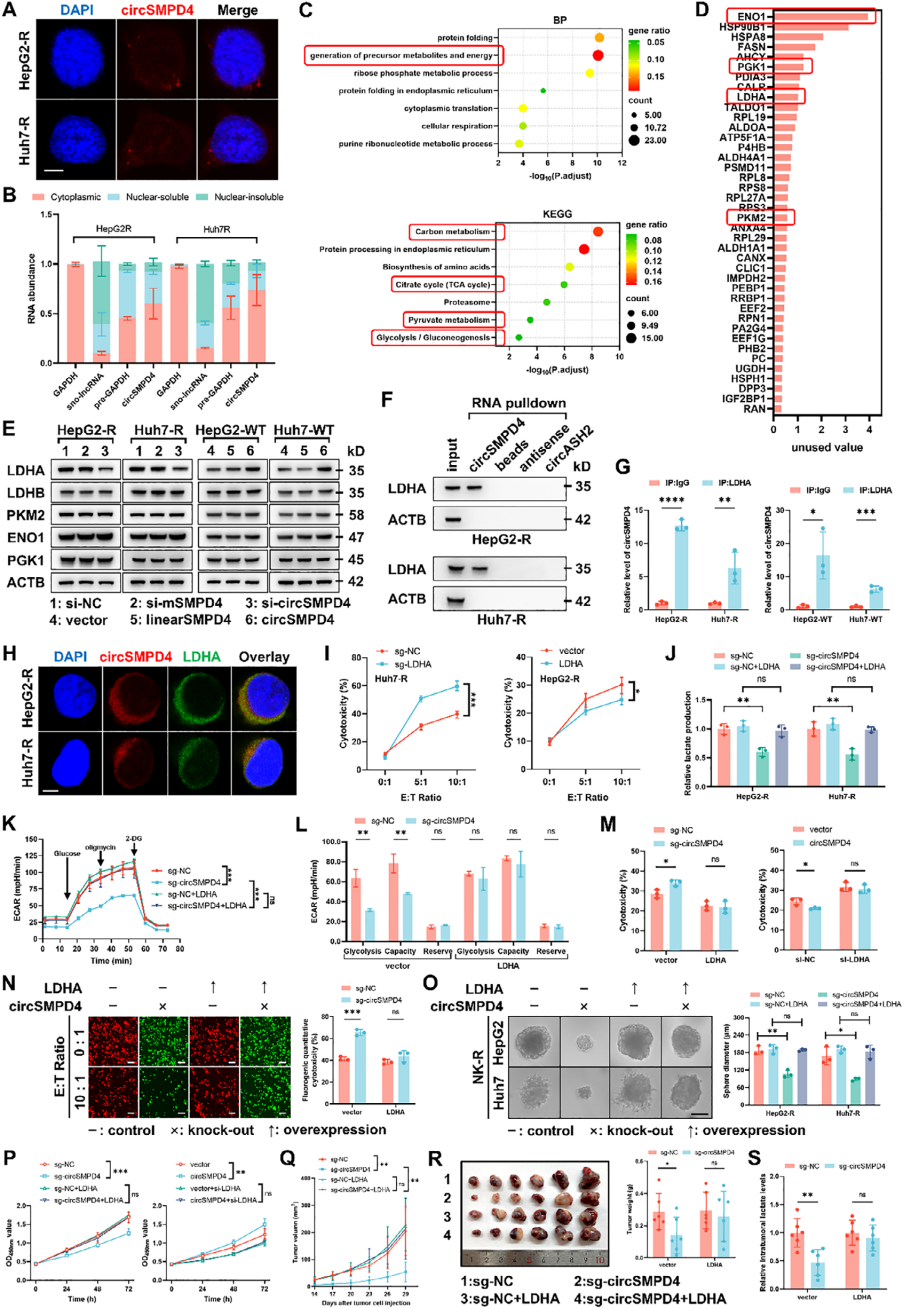
The circSMPD4/LDHA axis mediates circSMPD4's biological functions. A) Subcellular localization of circSMPD4 (red) in HepG2‐R and Huh7‐R cells revealed by FISH. Nuclei were labeled with DAPI (blue). Scale bars, 5 µm. B) RT‐qPCR detection of circSMPD4 from the indicated compartments of HepG2‐R and Huh7‐R cells, *n* = 3 independent biological replicates per group. GAPDH mRNA, sno‐lncRNA, and pre‐GAPDH mRNA were used as reference RNAs from the cytoplasm, nuclear‐insoluble component, and nuclear‐soluble component, respectively. C) Biological process (BP) in gene ontology (GO) analysis and Kyoto Encyclopedia of Genes and Genomes (KEGG) pathway enrichment analysis of circSMPD4‐interacting proteins. Glucose‐metabolism‐related terms are marked by red box. D) A diagram showing potential circSMPD4‐binding proteins identified by MS (listed in order of unused value). Key enzymes of glycolysis and lactate metabolism are marked by red box. E) Tumor cells with circSMPD4 knockdown or overexpression were analyzed for LDHA expression and other related metabolic enzymes by immunoblotting with the indicated antibodies. ACTB was used as an internal reference. F) LDHA was confirmed as circSMPD4‐binding protein by immunoblot analysis in HepG2‐R and Huh7‐R cells. G) Endogenous LDHA coimmunoprecipitated with endogenous circSMPD4 as determined by RT‐qPCR, *n* = 3 independent biological replicates per group (mean ± SD, *p < 0.05, **p < 0.01, ***p < 0.001, ****p < 0.0001, unpaired Student's *t*‐test). H) RNA‐FISH and immunostaining showing colocalization of circSMPD4 and LDHA in HepG2‐R and Huh7‐R cells. Scale bars, 4 µm. I) NK‐R cells with LDHA knockdown or overexpression were subjected to NK cell cytotoxicity assays at various E:T ratios using flow cytometry, *n* = 3 independent biological replicates per group (*p < 0.05, ***p < 0.001, two‐way ANOVA test). J) Reexpression of LDHA rescued the function of circSMPD4 on lactate production, *n* = 3 independent biological replicates per group (**p < 0.01, n.s., not significant, unpaired Student's *t*‐test). K,L) Overexpression of LDHA abolished the differences on extracellular acidification rate, *n* = 4 independent biological replicates per group (K, ***p < 0.001, n.s., not significant, two‐way ANOVA test) and glycolysis ability (L, **p < 0.01, n.s., not significant, unpaired Student's *t*‐test) with circSMPD4 knockout. M) Reexpression of LDHA restored circSMPD4‐mediated inhibition on NK cell cytotoxicity using flow cytometry, while knockdown of LDHA markedly revoked the function of circSMPD4, *n* = 3 independent biological replicates per group (*p < 0.05, n.s., not significant, unpaired Student's *t*‐test). N,O) LDHA rescued circSMPD4 induced phenotypes in fluorescent‐labeled NK cell cytotoxicity assays, *n* = 3 independent biological replicates per group (N, left: representative fluorescence images were shown, scale bar, 100 µm, right: quantification of NK cell cytotoxicity, ***p < 0.001, n.s., not significant, unpaired Student's *t*‐test) and in vitro 3D invasion assays (O, left: representative images were shown, scale bar, 100 µm, right: the diameter of tumor sphere was measured, *p < 0.05, **p < 0.01, n.s., not significant, unpaired Student's *t*‐test). P) CCK‐8 assays with indicated groups, OD_450nm_ value were detected every 24 h, *n* = 3 independent biological replicates per group (**p < 0.01, ***p < 0.001, two‐way ANOVA test). Q) Tumor volume trends for each group were represented by distinct colored curves, *n* = 5 mice per group (**p < 0.01, n.s., not significant, two‐way ANOVA test). R) Orthotopic tumors of indicated groups were isolated from mouse liver (left) and the weight of each tumor was quantified (right), *n* = 6 mice per group (mean ± SD. *p < 0.05, unpaired Student's *t*‐test). S) Reexpression of LDHA restored intratumoral levels of lactate in HepG2‐R circSMPD4‐knockout tumors, *n* = 6 mice per group (mean ± SD, **p < 0.01, n.s., not significant, unpaired Student's *t*‐test).

To elucidate the underlying mechanism of circSMPD4's effects, we first determined its subcellular localization. Fluorescence in situ hybridization (FISH) and nuclear–cytoplasm fractionation experiments revealed predominant cytoplasmic localization of circSMPD4 across various HCC cell lines, despite its intronic origin (**Figure** **4**A,B). Argonaute2 RNA immunoprecipitation (RIP) assays excluded the classical “miRNA sponge” function for circSMPD4 (Figure S4A, Supporting Information), and we confirmed its lack of protein‐coding capacity (Figure S4B, Supporting Information). Given the frequently uncovered role of circRNAs as protein scaffolds, we enriched circSMPD4 via circRNA pull‐down assay and employed mass spectrometry (MS) analysis to identify potential binding partners of circSMPD4. Functional enrichment analysis of interacting proteins indicated a strong association with carbon metabolism (Figure 4C; Figure S4C, Supporting Information), corroborating our previous metabolism‐related findings with circSMPD4. Notably, several key enzymes in glycolysis and lactate metabolism, including ENO1, PGK1, LDHA, and PKM2, were identified in the top circSMPD4 binding protein list (Figure 4D). Immunoblotting results demonstrated that circSMPD4 specifically modulates LDHA expression levels, while having no effect on the expression of several other glycolytic enzymes (Figure 4E). With previous data showing that circSMPD4 affects lactate production rather than pyruvate (Figure 3J; Figure S3J, Supporting Information), we suspected that LDHA might be a vital effector protein of circSMPD4. Subsequent validation through circRNA pull‐down immunoblotting (Figure 4F), RIP assay (Figure 4G), and fluorescence colocalization staining (Figure 4H) confirmed a significant interaction exclusively between circSMPD4 and LDHA. Furthermore, LDHA expression was found to negatively correlate with HCC prognosis (Figure S4D, Supporting Information). LDHA, the main subunit of key enzyme for lactate metabolism, catalyzes the reversible conversion of pyruvate to lactate. Apparently, LDHA expression levels positively correlated with lactate production (Figure S4E, Supporting Information). Flow cytometry analysis of NK cell cytotoxicity assays revealed that LDHA knockout significantly reduced tumor cell resistance to NK‐cell‐mediated cytotoxicity, while LDHA overexpression yielded opposite results (Figure 4I).

### 2.7 CircSMPD4's Biological Function Is LDHA‐Dependent

To further establish LDHA's indispensable role in circSMPD4's functional processes, we conducted a series of experiments using LDHA‐manipulated cell lines. We engineered tool cell lines with LDHA overexpression/knockdown, confirming specific modulation of LDHA expression levels through immunoblotting (Figure S4F, Supporting Information). Notably, LDHA overexpression efficiency was limited, potentially due to high basal LDHA expression in tumor cell lines, which may explain the limited effect of circSMPD4 overexpression on LDHA levels. Moreover, LDHA overexpression enhanced lactate production in tumor cells, completely offsetting the effects of circSMPD4 knockout, while LDHA knockdown in circSMPD4‐overexpressing cells yielded similar conclusion (Figure 4J; Figure S4G, Supporting Information). LDHA also eliminated circSMPD4's effects on cellular ECAR (Figure 4K,L; Figure S4H,I, Supporting Information). In vitro NK cell cytotoxicity assays demonstrated that LDHA could reverse circSMPD4‐induced NK‐cell‐mediated immune evasion (Figure 4M,N; Figure S4J, Supporting Information). Flow cytometry and RT‐qPCR revealed that LDHA could abolish circSMPD4's indirect suppressive effect on NK cell cytotoxicity (Figure S4K,L, Supporting Information). Furthermore, LDHA proved essential for circSMPD4‐mediated promotion of tumor invasion and proliferation (Figure 4O,P; Figure S4M, Supporting Information). For in vivo assays, we first demonstrated that LDHA level was markedly reduced in circSMPD4 knockout tumor (Figure S4N, Supporting Information). LDHA overexpression markedly rescued the reduced tumor volume caused by circSMPD4 knockout (Figure 4Q,R). Intratumoral lactate measurements confirmed that LDHA overexpression could reverse the low lactate concentration in the TME induced by circSMPD4 knockout (Figure 4S). The above results indicated that the upregulation of LDHA by circSMPD4 not only induced NK‐cell‐mediated tumor immune evasion but also enhanced the malignant biological behaviors of HCC.

Collectively, these data establish LDHA as a core effector for circSMPD4's biological functions, indispensable for circSMPD4‐promoted tumor immune evasion and tumor progression.

### 2.8 CircSMPD4 Inhibits LDHA Degradation via CMA Pathway

**Figure 5:**
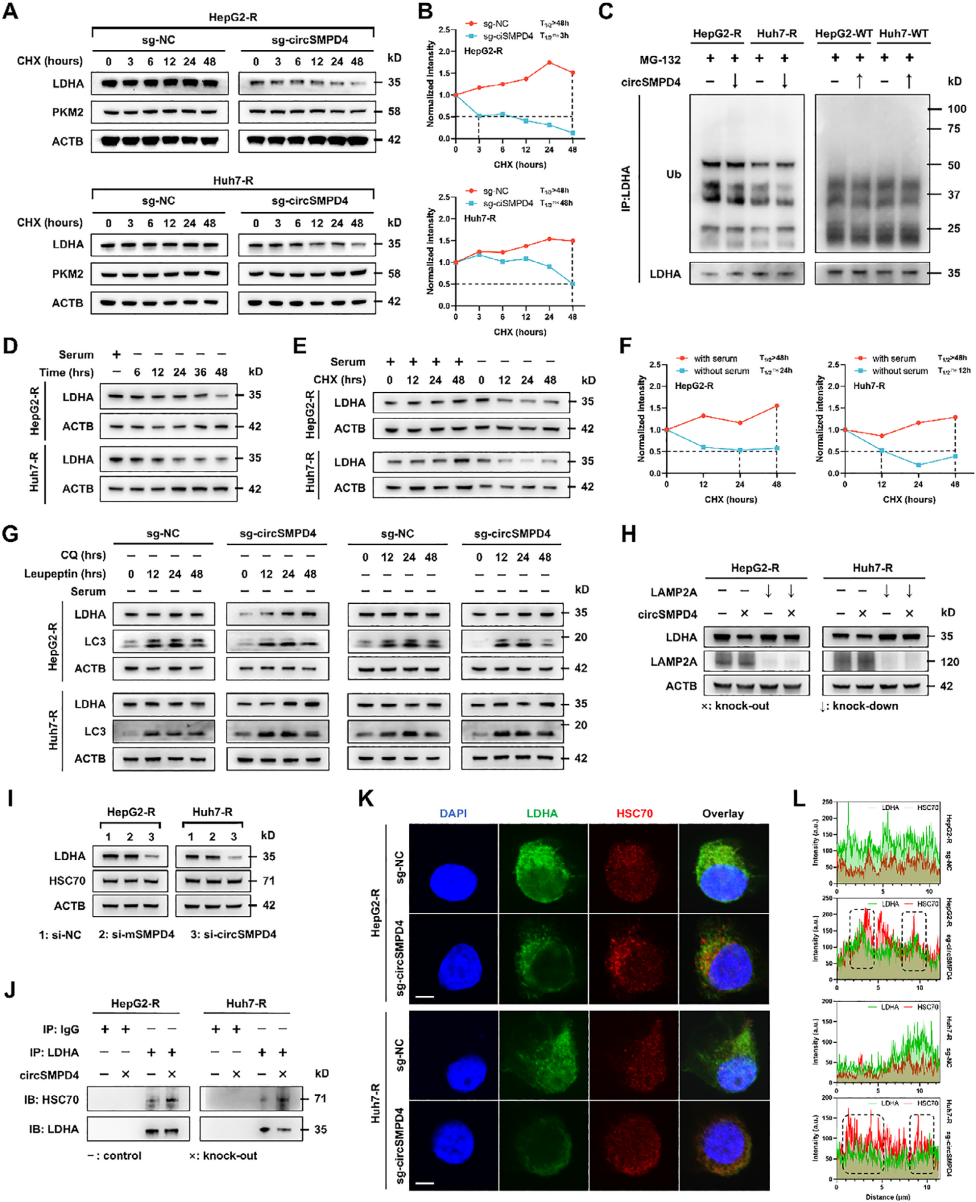
CircSMPD4 inhibits the CMA‐mediated degradation of LDHA. A,B) HepG2‐R or Huh7‐R cells ± circSMPD4 knockout were treated with CHX (5.0 µg mL^−1^) as indicated. Endogenous LDHA was determined by immunoblotting (A) and quantified (B). The dashed line shows the half‐life of LDHA. PKM2 was used as a negative control. C) The ubiquitination level of endogenous LDHA was extremely low, and circSMPD4 did not affect the ubiquitination level of LDHA. D) Serum deprivation decreased LDHA level in tumor cells. LDHA level was determined by immunoblotting at indicated time points after serum deprivation. E,F) LDHA suffered an accelerated degradation after serum deprivation in tumor cells. Tumor cells were treated with CHX (5.0 µg mL^−1^) in the presence of serum or not. LDHA level was determined at indicated time points by immunoblotting (E) and quantified (F). The dashed line shows the half‐life of LDHA. G) Tumor cells ± circSMPD4 knockout were treated with CQ (20 µm) or leupeptin (50 µm) under the condition of serum deprivation. LDHA and LC3 levels were determined at indicated time points by immunoblotting. H) LAMP2A knockdown blocked the effect of circSMPD4‐knockout on LDHA levels. The level of LDHA was determined by immunoblotting. I) The levels of LDHA and HSC70 in tumor cells ± circSMPD4/mSMPD4 knockdown were determined by immunoblotting. J) Endogenous LDHA was immunoprecipitated in tumor cells ± circSMPD4 knockout. The levels of HSC70 and LDHA were determined by immunoblotting. K,L) Increasing colocalization of HSC70 and LDHA after circSMPD4 knockout was showed by immunostaining (K) and quantified (L, quantification of immunofluorescence was performed using ImageJ. The analysis was conducted within the region corresponding to the lengthwise dimension of the cell. Peaks indicated of significant colocalization are marked with black dashed lines). Scale bars, 5 µm.

To further explore the mechanism by which circSMPD4 modulates LDHA expression, we conducted a series of experiments. Given that LDHA regulation may occur at transcriptional or posttranscriptional levels, we first demonstrated that circSMPD4 does not affect LDHA transcription (Figure S5A, Supporting Information). Subsequently, we focused on protein‐level regulation, particularly protein degradation, as it is a common posttranscriptional regulation mechanism. Using the ribosome inhibitor cycloheximide (CHX) to block LDHA protein synthesis, we observed that circSMPD4 knockout significantly accelerated LDHA protein degradation and shortened its half‐life (**Figure** **5**A,B). Our investigation then centered on identifying the specific protein degradation pathway involved in circSMPD4's protection of LDHA. The two main protein degradation pathways in eukaryotic cells are lysosomal and proteasomal, with additional nonclassical pathways. However, the specific degradation pathway for LDHA remains inconclusive. Interestingly, we observed that LDHA in control HCC cells did not undergo significant degradation within 24 h of CHX treatment (Figure 5A,B). This aligns with previous studies suggesting LDHA is a long‐lived protein with an extended degradation half‐life.^[^ ^49^ ^]^ Consequently, we extended the observation period for LDHA protein level changes in subsequent experiments to better characterize its degradation kinetics and the impact of circSMPD4 on this process.

Protein degradation via the classical proteasomal pathway requires ubiquitin tagging, a process catalyzed by E1 ubiquitin‐activating enzyme (UAE), E2 ubiquitin‐conjugating enzymes, and E3 ubiquitin ligase enzymes. Recently, a novel study has implicated that the specific phosphorylation of LDHA at certain sites could inhibit its ubiquitination in the nucleus.^[^ ^50^ ^]^ To investigate whether circSMPD4's regulation of LDHA persists when ubiquitination is blocked, we employed selective UAE inhibitors PYR‐41 and TAK‐243. Using known ubiquitination‐dependent degradation proteins (p27) as positive controls, we established that under normal conditions, LDHA in both WT and NK‐R cells was not degraded through the ubiquitination‐proteasome pathway (Figure S5B, Supporting Information). Neither UAE inhibitor rescued the decrease in LDHA levels caused by circSMPD4 knockout (Figure S5C, Supporting Information). Immunoprecipitation assay revealed limited LDHA ubiquitination, unaffected by circSMPD4 knockout (Figure 5C). Recent research has identified midnolin, an inducible nuclear‐localized protease that promotes nonubiquitinated transcriptional regulator degradation.^[^ ^51^ ^]^ Building on our previous discovery of a circRNA‐mediated, ubiquitination‐independent proteasomal degradation pathway,^[^ ^38^ ^]^ we hypothesized a similar role for circSMPD4. However, the proteasome inhibitor MG‐132 failed to reverse LDHA downregulation induced by circSMPD4 knockout (Figure S5D, Supporting Information), indicating that circSMPD4‐induced LDHA degradation is independent of the proteasomal pathway.

We then investigated the lysosomal degradation pathway, encompassing macroautophagy, microautophagy, and CMA. Surprisingly, under standard culture conditions, lysosomal inhibitors chloroquine (CQ) and leupeptin did not reverse LDHA downregulation induced by circSMPD4 knockout (Figure S5E, Supporting Information). However, a seminal study suggested that autophagy participates in protein quality control under stress conditions.^[^ ^52^ ^]^ We hypothesized that specific stress conditions might trigger circSMPD4‐knockout‐induced LDHA downregulation. Starvation conditions are known to activate intracellular autophagy.^[^ ^53^ ^]^ Indeed, we observed decreased LDHA expression in tumor cells under serum‐free culture conditions (Figure 5D), with accelerated LDHA degradation after cycloheximide treatment (Figure 5E,F). Under starvation conditions, lysosomal inhibitors reversed LDHA downregulation caused by circSMPD4 knockout (Figure 5G). Notably, an authoritative study had reported that LDHA can be degraded through the CMA‐lysosomal pathway in pancreatic cancer.^[^ ^54^ ^]^ CMA involves the molecular chaperone heat shock cognate 71 kDa protein (HSC70/HSPA8) recognizing and binding a specific motif on substrate proteins, facilitating their transport into lysosomes for degradation, while LAMP2A plays a crucial role in regulating CMA flux.^[^ ^55^ ^]^ shRNA‐mediated LAMP2A silencing reversed LDHA downregulation caused by circSMPD4 knockout (Figure 5H), supporting our hypothesis. We confirmed that circSMPD4 had no effect on HSC70 expression levels (Figure 5I). Coimmunoprecipitation experiments demonstrated that circSMPD4 significantly inhibited the physical binding between LDHA and HSC70 (Figure 5J). Immunofluorescence experiments corroborated these findings, showing increased colocalization of LDHA and HSC70 in circSMPD4 knockout cells (Figure 5K,L). In conclusion, these results provide compelling evidence that circSMPD4 inhibits LDHA degradation through the CMA pathway by preventing LDHA─HSC70 binding, thus providing a novel mechanism of circRNA‐mediated protein stabilization.

### 2.9 CircSMPD4 Inhibits LDHA Degradation via CMA‐Lysosomal Pathway by Promoting SIRT2‐Mediated Deacetylation of LDHA

**Figure 6:**
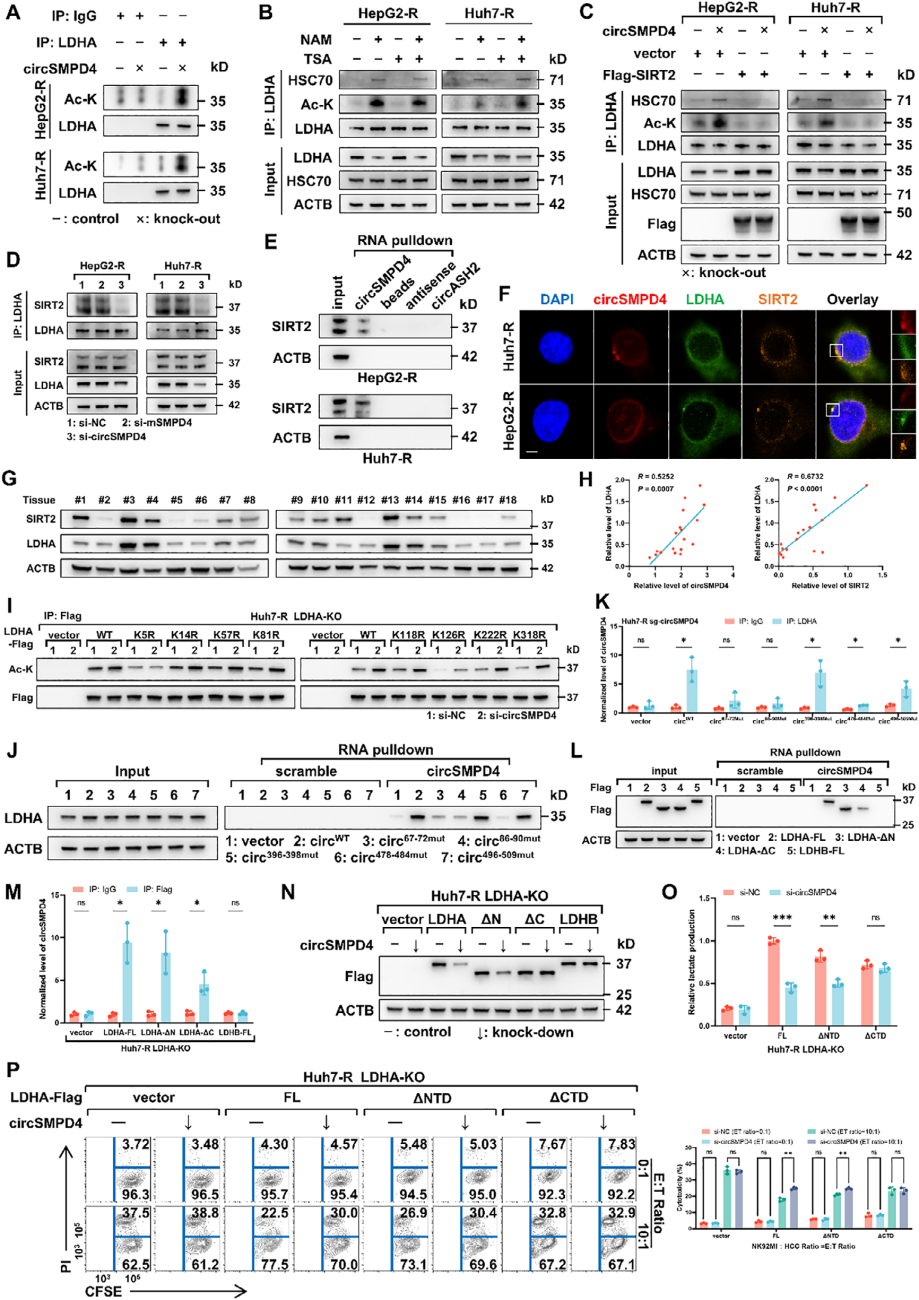
CircSMPD4 promotes SIRT2‐mediated deacetylation of LDHA and its interaction with C‐terminus of LDHA is vital for biological function. A) Endogenous LDHA was immunoprecipitated in tumor cells ± circSMPD4 knockout. LDHA acetylation and protein levels were determined by immunoblotting. B) Tumor cells were treated with deacetylase inhibitors TSA, NAM, or together for 24 h and then endogenous LDHA was immunoprecipitated. LDHA acetylation, protein levels of LDHA and HSC70 were determined by immunoblotting. C) Overexpression of SIRT2 markedly rescued the downregulation and acetylation of LDHA caused by circSMPD4 knockout, sharply blocking the combination between LDHA and HSC70. LDHA acetylation and protein levels were determined by immunoblotting with indicated antibody. D) Knockdown of circSMPD4, not mSMPD4, impaired the combination between LDHA and SIRT2. Endogenous LDHA was immunoprecipitated in HCC NK‐R cells ± circSMPD4/mSMPD4 knockdown. LDHA and SIRT2 levels were determined by immunoblotting. E) Detection of endogenous SIRT2 following circSMPD4 pull‐down from HepG2‐R and Huh7‐R cells, while circASH2 was used as a negative control. F) RNA‐FISH and immunostaining showing colocalization of circSMPD4, LDHA, and SIRT2 in NK‐R cells. Scale bars, 5 µm. G) Protein levels of LDHA and SIRT2 in HCC tissues (cohort 1) were determined by immunoblotting. ACTB was used as an internal reference. H) Correlations between expression levels of circSMPD4 and LDHA (left), SIRT2 and LDHA (right) in HCC tissues (cohort 1). RNA levels were determined using RT‐ qPCR and normalized to ACTB. The *R* values and *p* values were calculated using Pearson correlation analysis. I) The indicated plasmids of individual LDHA mutants were transfected into Huh7‐R sg‐LDHA cells. Immunoblots analysis was used to detect the acetylation level of immunoprecipitated protein. J) Huh7‐R sg‐circSMPD4 cells were transduced with circSMPD4^WT^, circSMPD4^67–72mut^, circSMPD4^86–90mut^, circSMPD4^396–398mut^, circSMPD4^478–484mut^, and circSMPD4^496–509mut^, respectively. Immunoblots analysis was used to detect the endogenous LDHA enriched by circSMPD4 pull‐down. K) Huh7‐R sg‐circSMPD4 cells were transduced with circSMPD4^WT^, circSMPD4^67–72mut^, circSMPD4^86–90mut^, circSMPD4^396–398mut^, circSMPD4^478–484mut^, and circSMPD4^496–509mut^, respectively. RT‐qPCR was used to detect the circSMPD4 enriched by anti‐LDHA immunoprecipitation, *n* = 3 independent biological replicates per group (*p < 0.05, n.s., not significant, unpaired Student's *t*‐test). L) LDHA‐KO Huh7‐R cells were transduced with LDHA^FL^‐3×Flag, LDHA^ΔNTD^‐3×Flag, LDHA^ΔCTD^‐3×Flag, and LDHB^FL^‐3×Flag, respectively. The level of LDHA‐3×Flag enriched by circSMPD4 pull‐down was determined by immunoblotting. M) RNA immunoprecipitation assays with endogenous circSMPD4 as determined by RT‐qPCR in indicated cells, *n* = 3 independent biological replicates per group (mean ± SD, *p < 0.05, n.s., not significant, unpaired Student's *t*‐test). N) LDHA‐KO Huh7‐R cells were reconstituted with LDHA^FL^, LDHA^ΔNTD^, LDHA^ΔCTD^, and LDHB^FL^, respectively. The level of LDHA‐3×Flag in indicated cells was determined by immunoblotting. O) Measurement of supernatant levels of lactate in indicated cells, *n* = 3 independent biological replicates per group. LDHA‐KO Huh7‐R cells were reconstituted with LDHA^FL^, LDHA^ΔNTD^, and LDHA^ΔCTD^, respectively (mean ± SD, **p < 0.01, ***p < 0.001, n.s., not significant, unpaired Student's *t*‐test). P) LDHA‐KO Huh7‐R cells were reconstituted with LDHA^FL^, LDHA^ΔNTD^, and LDHA^ΔCTD^, respectively. Indicated cells were subjected to NK cell cytotoxicity assay at various E:T ratios using flow cytometry. Representative images were shown (left) and results were quantified, *n* = 3 independent biological replicates per group (right, **p < 0.01, n.s., not significant, unpaired Student's *t*‐test).

Previous studies have indicated that acetylation modifications of LDHA can promote its CMA‐mediated degradation,^[^ ^54^ ^]^ thus we intend to investigate whether circSMPD4 influences the acetylation of LDHA. Immunoprecipitation experiments revealed that circSMPD4 knockout significantly increased overall LDHA lysine acetylation (**Figure** **6**A).

It is well‐established that acetylation is a widespread, reversible posttranslational protein modification, primarily regulated by two classes of enzymes: lysine acetyltransferases (KATs) and lysine deacetylases (KDACs). KATs catalyze the transfer of acetyl groups to lysine residues, while KDACs catalyze the removal of acetyl groups. KATs have been reported to primarily use histones as substrates and exert their catalytic activity predominantly in the nucleus.^[^ ^56^ ^]^ Given that LDHA is a cytoplasmic, nonhistone protein, we hypothesized that its acetylation level might be more dependent on KDACs than KATs. We focused on two KDAC subgroups: classical Zn^2+^‐dependent histone deacetylases (HDACs I, II, IV) and NAD+‐dependent deacetylases (SIRT family). Treatment with deacetylase inhibitors trichostatin A (TSA, HDAC I and II inhibitor) and nicotinamide (NAM, SIRT family inhibitor) revealed that NAM, rather than TSA, could increase LDHA acetylation levels and facilitate the physical binding between LDHA and HSC70, leading to a significant downregulation of LDHA (Figure 6B). Among the SIRT family, SIRT2 primarily functions in the cytoplasm and has been reported to use LDHA as a substrate.^[^ ^54^, ^56^ ^]^ We confirmed that SIRT2 knockdown remarkably increased LDHA acetylation level while SIRT2 overexpression had opposite effects (Figure S6A,B, Supporting Information). Moreover, SIRT2 overexpression reversed LDHA downregulation and reduced LDHA─HSC70 combination induced by circSMPD4 knockout, while SIRT2 knockdown eliminated circSMPD4's function (Figure 6C; Figure S6C, Supporting Information). Additionally, we found that circSMPD4 did not affect SIRT2 protein levels but facilitate the physical binding between SIRT2 and LDHA (Figure 6D). CircRNA pull‐down assay that confirmed a physical interaction between circSMPD4 and SIRT2 (Figure 6E), and immunofluorescence experiments showed a clear colocalization among circSMPD4, LDHA, and SIRT2 (Figure 6F). Taken together, we considered that circSMPD4 might serve as a protein scaffold to stabilize the combination between LDHA and SIRT2. Further analysis of HCC cohort from TCGA database revealed that SIRT2 expression levels were not significantly correlated with the prognosis of HCC patients (Figure S6D, Supporting Information). To validate the functional role of circSMPD4, we analyzed the relationship among circSMPD4, LDHA, and SIRT2 expression levels in clinical HCC samples. Specifically, we assessed the protein levels of LDHA and SIRT2 in 18 HCC tissues from cohort 1 (Figure 6G). In combination with our previously obtained circSMPD4 expression data (Figure 1J), we found that both circSMPD4 and SIRT2 expression levels were significantly positively correlated with LDHA (Figure 6H), whereas no notable correlation was observed between circSMPD4 and SIRT2 (Figure S6E, Supporting Information). Analysis of the HCC cohort from the TCGA dataset further revealed a positive correlation between the expression levels of SIRT2 and LDHA (Figure S6F, Supporting Information). To further identify the critical site through which circSMPD4 influences LDHA acetylation, we generated LDHA‐knockout tumor cell lines (HepG2‐R LDHA‐KO and Huh7‐R LDHA‐KO) (Figure S6G, Supporting Information) and referenced the mass spectrometry results from an outstanding study. This mass spectrometry analysis identified eight putative acetylation sites on LDHA.^[^ ^57^ ^]^ We mutated each of these eight sites to arginine and subsequently discovered that mutations at K5, K126 or K318 (but not other lysine residues) led to a significant reduction in LDHA acetylation, while only the K5 mutation resulted in the loss of circSMPD4 function (Figure 6I). Therefore, we conclude that K5 is a critical site for circSMPD4‐mediated reduction of LDHA acetylation.

### 2.10 CircSMPD4's Interaction with C‐Terminus of LDHA Is Crucial for Its Biological Function

To elucidate the detailed mechanism, we employed advanced molecular structural biology techniques. We obtained circSMPD4's secondary structure at its lowest free energy using UNAFold software (Figure S6H, Supporting Information) and constructed its tertiary structure using 3dRNA (Figure S6I, Supporting Information). We then simulated the circSMPD4─LDHA interaction using HDOCK software, based on the LDHA model from UniProt (Figure S6J, Supporting Information). Interaction simulation analysis revealed the putative binding sites between circSMPD4 and LDHA (Figure S6K and Table S2, Supporting Information). Further identifying of the circSMPD4 mutant fragment indicated that 67–72, 86–90, and 478–484 nt regions of circSMPD4 were indispensable for binding to LDHA (Figure 6J,K). To further detail the interaction mechanism, we reexpressed LDHA FL‐Flag, LDHA ΔNTD‐Flag, LDHA ΔCTD‐Flag, and LDHB FL‐Flag in LDHA‐KO cell lines (Figure S6L, Supporting Information). CircRNA pull‐down and RNA immunoprecipitation experiments confirmed the importance of the CTD fragment for circSMPD4 binding (Figure 6L,M; Figure S6M,N, Supporting Information). We then investigated the CTD fragment's essentiality for circSMPD4 function. Immunoblotting revealed that the CTD fragment was necessary for circSMPD4‐mediated regulation of LDHA expression (Figure 6N). Functional assays demonstrated that only the absence of the CTD fragment impaired circSMPD4's ability to promote lactate production in tumor cells (Figure 6O; Figure S6O, Supporting Information). NK cell cytotoxicity assays and 3D invasion assays further confirmed the CTD fragment's crucial role in circSMPD4's biological functions (Figure 6P; Figure S6P,Q, Supporting Information).

In conclusion, these findings elucidate a novel mechanism whereby circSMPD4 physically interacts with LDHA, promoting the deacetylation of LDHA via SIRT2 and consequently protecting its degradation from the CMA‐lysosome pathway. This interaction between circSMPD4 and LDHA CTD is critical for circSMPD4's diverse biological functions in HCC.

## 3 Discussion

Tumor‐evolution‐induced heterogeneity and increased malignancy represent crucial factors contributing to poor clinical outcomes in malignant tumors.^[^ ^10^, ^11^, ^12^ ^]^ In our previous work, we established a model of tumor evolution under sustained NK cell immunosurveillance pressure. Recent high‐quality studies have consistently demonstrated the intimate connection between circRNAs and cancer, with researchers increasingly acknowledging the significant roles of specific circRNAs in various cancers.^[^ ^32^ ^]^ In this study, through public databases and our established NK‐cell‐driven tumor evolution model, we innovatively identified a previously unreported oncogenic circRNA—circSMPD4—showing gradual upregulation from normal liver tissue to LI HCC tissue to HI HCC tissue. Through various in vivo and in vitro HCC models, we comprehensively demonstrated that circSMPD4 promotes tumor lactate metabolic reprogramming by inhibiting LDHA degradation through CMA‐lysosome pathway, ultimately inducing NK‐cell‐related immune escape and enhancing HCC malignancy.

As widely recognized, NK cell is the core effector cell of innate immune responses. Compared to other organs, the notable enrichment of NK cells in liver makes them equally crucial in hepatic antipathogen and antitumor processes,^[^ ^58^, ^59^ ^]^ which has become a new direction for immunotherapy research.^[^ ^40^, ^60^, ^61^ ^]^ Importantly, NK cells have been proven to participate in tumor immunity from early stages and play vital roles during tumor progression.^[^ ^13^ ^]^ Additionally, NK cell quantity, infiltration rate, and functional status in TIME have been demonstrated to directly influence HCC patient prognosis.^[^ ^9^ ^]^ However, the intrinsic mechanisms of NK‐cell‐related immune escape in cancer research, particularly in HCC, remain unclear. Notably, recent studies suggest that circRNAs can regulate NK cell immunosurveillance capacity. For instance, circSOBP in glioma enhances NK cell killing function through the IKKε/TBK1/IRF3 signaling pathway by promoting interferon expression.^[^ ^62^ ^]^ Furthermore, circUHRF1 in HCC exosomes has been shown to suppress NK cell IFN‐γ secretion, thereby weakening their antitumor activity.^[^ ^63^ ^]^ In our study, we found that circSMPD4 does not affect the expression of NK‐cell‐related activating and inhibitory ligands on tumor cell surface, but rather indirectly inhibits NK cell cytotoxicity by increasing lactate production and export. Numerous studies have shown that tumor cells can adapt to TME characterized by metabolic nutrient scarcity (e.g., glucose, glutamine) and metabolic waste abundance (e.g., lactate) through metabolic reprogramming, while such TME often strongly suppresses cytotoxic immune cells.^[^ ^14^, ^15^, ^16^ ^]^ Predictably, deeply analyzing the molecular mechanisms of suppressed NK cell immunosurveillance in HCC TIME and attempting to activate their potential antitumor activity represents an urgent scientific issue in future HCC immunotherapy research.

CircRNA potential functions and mechanisms are closely related to their subcellular localization.^[^ ^64^ ^]^ Previous studies suggest that exonic circRNAs primarily localize in the cytoplasm, while intron‐containing circRNAs tend to distribute in the nucleus.^[^ ^65^ ^]^ Among these, intron‐containing circRNAs are mostly reported to regulate transcription of parental or nonparental genes in the nucleus.^[^ ^66^, ^67^ ^]^ Currently, particularly in cancer research, intronic circRNAs are rarely reported to execute specific biological functions in the cytoplasm. In our study, circSMPD4, as an intronic circRNA formed by a single intron, was found to primarily localize in tumor cell cytoplasm and perform specific biological function. However, how circSMPD4 exports from nucleus to cytoplasm warrants further investigation. Notably, recent high‐quality research has shown that IGF2BP1 can directly bind to nuclear circRNAs and recruit Ran‐GTP and exportin‐2 to form circRNA export complexes facilitating circRNA nuclear export.^[^ ^68^ ^]^ According to Chen et al.’s research, *N*6‐methyladenosine modification of circNSUN2 promotes its nuclear export to cytoplasm and stabilizes HMGA2 to promote colorectal cancer liver metastasis.^[^ ^69^ ^]^ One study related to bladder cancer reported that SUMOylated DDX39B activated the nuclear export of circNCOR1,^[^ ^70^ ^]^ while another notable study revealed that adenosine‐rich circRNAs were retained in the H9 nucleus via nuclear PABPC1 and the nuclear basket protein TPR, with the loss of constraint by PABPC1 leading to their export during differentiation.^[^ ^71^ ^]^ These significant findings suggest that the export of circRNA from the nucleus likely requires the formation of a circRNA export complex through its association with a specific circRNA transport protein. It is conceivable that a specific protein may serve as the transport mediator for circSMPD4, facilitating its nuclear export. However, further investigations are essential to fully elucidate the underlying mechanisms. Overall, our study unveils a novel intronic circRNA exhibiting nonclassical specific oncogenic functions in the cytoplasm, providing new insights for in‐depth study of circRNA–cancer‐related mechanisms.

The dynamic balance between protein synthesis and degradation is crucial for maintaining cellular homeostasis, with eukaryotic protein degradation metabolism primarily accomplished through lysosomal or proteasomal systems.^[^ ^72^ ^]^ In this study, we found that circSMPD4 presence exhibits strong “protective effects” on LDHA levels, and subsequent data revealed that circSMPD4 does not affect LDHA transcription levels but LDHA degradation half‐life. Like most metabolic enzymes, LDHA is a highly stable protein, with high‐level research reporting its degradation half‐life far exceeding 8 h,^[^ ^49^ ^]^ which was also confirmed in our study. An authoritative study has previously demonstrated that autophagy is involved in protein quality control under stress conditions,^[^ ^52^ ^]^ and serum deprivation has been reported to activate autophagy.^[^ ^53^ ^]^ Consistently, we observed a remarkable degradation of LDHA under serum deprivation condition, along with markedly inhibitory effect of circSMPD4 on LDHA degradation. Previous studies on LDHA degradation's intrinsic mechanisms lack definitive conclusions, while one outstanding study reported that elevated LDHA acetylation at lysine 5 leads to its degradation via CMA‐lysosome pathway and SIRT2 is the deacetylase of LDHA.^[^ ^54^ ^]^ CMA is a highly substrate‐specific form of autophagy requiring chaperone protein HSC70 recognition and binding to specific sequences on substrate proteins.^[^ ^55^ ^]^ Our data suggest that circSMPD4 functions as a protein scaffold for LDHA and SIRT2, thereby enhancing the deacetylation of LDHA by SIRT2 and inhibiting its lysosomal degradation mediated by the binding of LDHA to HSC70. Importantly, the analysis of TCGA HCC cohort confirmed that SIRT2 expression levels were not significantly associated with HCC prognosis. We believe that this lack of correlation may be due to the broader enzymatic role of SIRT2 as a deacetylase for multiple biological enzymes. Overall, its expression level does not show a significant relationship with HCC prognosis. Our results demonstrated that circSMPD4 specifically enhances SIRT2‐mediated deacetylation of LDHA through specific binding to both LDHA and SIRT2, rather than altering the expression levels of SIRT2, and SIRT2 is necessary for circSMPD4's function.

In summary, this study addresses current hot research directions including tumor metabolic reprogramming, tumor immune evasion, and circRNA. Focusing on HCC with poor immunotherapy efficacy, against the background of NK‐cell‐induced HCC tumor evolution, we investigated the intrinsic mechanisms through which circSMPD4 affects tumor metabolic reprogramming, immune escape, and progression from the perspective of circRNAs, providing valuable insights into poor clinical efficacy in HCC. In this project, circSMPD4, as a novel oncogenic circRNA exhibited a gradient increase of expression levels in adjacent normal tissues – LI HCC tissues – HI HCC tissues, induces tumor immune evasion and tumor progression by enhancing tumor cell Warburg effect. Mechanistically, circSMPD4 promotes SIRT2‐mediated LDHA deacetylation via physical combination with specific regions of LDHA, thereby preventing LDHA lysosomal degradation through CMA pathway. The unique functions executed by circSMPD4 in these mechanisms undoubtedly bring new inspiration to related research fields. In recent years, many single‐cell high‐throughput sequencing and proteogenomic analyses of HCC have provided key information about intrinsic changes in HCC tumor cells and their dynamic interactions with the tumor immune microenvironment, especially immune cells.^[^ ^73^, ^74^, ^75^, ^76^, ^77^ ^]^ Evidently, metabolic reprogramming is a key event in increased tumor cell malignancy, and changes in circRNA expression profiles and the corresponding functional effects undoubtedly provide a novel perspective, representing an important link in broadening our understanding in this field.

## 4 Experimental Section

### Patients

A total of 4 independent cohorts including 100 HCC patients were used in this study. Cohort 1 contained a group of 18 randomly selected HCC clinical samples collected from Sir Run‐Run Shaw Hospital starting in August 2018, which were used for detecting the expression levels of circSMPD4. Cohort 2 included 21 low and 11 highly invasive primary HCC samples collected from Sir Run‐Run Shaw Hospital starting in January 2018, which were used for verifying that the expression level of circSMPD4 was positively correlated with the malignancy of the tumor. Cohort 3 was a cohort of 23 liver cancer patients who underwent surgical treatment at the Sir Run Run Shaw Hospital (December 2021–February 2023) and was stratified into NK‐sensitive (NK‐S) or NK‐R groups. Primary tumor cells were isolated within 2 h postresection. Specimens with >50% viability by trypan blue staining were subjected to NK cell cytotoxicity assays and patients were classified as NK‐S or NK‐R group based on the cytotoxicity results. To control for batch effects and implement standardization procedures, NK92MI cells were simultaneously tested against both patient‐derived primary cells and HepG2 cells. Cohort 4 included 40 randomly selected HCC clinical samples collected from Sir Run‐Run Shaw Hospital starting in October 2013 with 5 years follow‐up information. Patients who succumbed to conditions unrelated to HCC or experienced unexpected events were excluded from the study cohort. The low and high circSMPD4 expressions were cutoff by median expression. This study conformed to the principles of the Declaration of Helsinki and was approved by the ethics committee of Sir Run Run Shaw Hospital, School of Medicine, Zhejiang University (2022‐604‐02). All patients signed informed consent before surgery for the use of their tissues for scientific research.

### Mice

The animal experimental protocols, conducted under license ZJU20240983, were approved by the ethics committee of the Zhejiang University. NCG mice were purchased from GemPharmatech (Nanjing, CN). HepG2‐R cells were engineered to express luciferase reporter gene by stable transfection, and the positive stable clones were selected with ampicillin and expanded in culture. After that, HCC (HepG‐2R and Huh7‐R) stable cell lines with circSMPD4 knockout or control vector were established. For orthotopic xenograft model, these cells were injected to NCG mice orthotopically to generate HCC tissues. After HCC tissues were prepared, prior to performing the liver orthotopic tumor implantation surgery, the mice with these primary HCC tissues were sacrificed. The tumors were carefully dissected, with particular attention given to the removal of necrotic or hemorrhagic areas. Viable tumor tissue was then cut into ≈2 mg pieces and rapidly implanted into the left lobe of the liver in both groups (control and circSMPD4‐knockout) of mice. For tail vein injection model of metastasis, mice (*n* = 7) were injected with control or circSMPD4‐knockout cells (1.5 × 10^6^ cells per mouse) through the tail vein. Metastasis was tracked by IVIS system mentioned before once every 2 weeks thereafter. Mice were housed in a fully enclosed barrier system with constant temperature, pressure, and humidity control.

### Reagents (e.g., Antibodies, Drugs, Proteins, Primers, Vectors, Etc.)

Detail of these reagents were included in the Supporting Information.

### Cell Culture

NK92MI cell was maintained in NK‐92MI cell‐specific culture medium (Procell, modified Eagle medium α (MEMα) + 0.2 mm inositol + 0.1 mm β‐mercaptoethanol + 0.02 mm folic acid + 12.5% HS + 12.5% fetal bovine serum (FBS) + 1% P/S). LO2, HepG2, HCCLM3 cells were maintained in Dulbecco's modified Eagle medium (Invitrogen) with 10% FBS (Cellmax), and 1% Glutamine. Huh7 cell was maintained in MEM (Invitrogen) with 10% FBS, and 1% Glutamine. All cells were cultured at 37 °C and 5% CO_2_. All cells were checked for mycoplasma by a PCR‐based method and DAPI staining to ensure the absence of contamination. Cell line sources were described in the Supporting Information.

### In Vitro Tumor Evolution Model

HCC WT cells were cocultured with NK92MI cells for 24 h, adjusting the effector‐to‐target (E:T) ratio to maintain ≈30% survival of tumor cells. The surviving tumor cells were allowed to recover and grow for 48–72 h, followed by a single passage. In parallel, control WT cells were discarded 70% of the cells randomly and passaged. Phenotypically stable HCC NK‐R cells were obtained after more than 20 cycles of stimulation.

### In Vitro Cytotoxicity Assay

HCC cells were labeled with carboxyfluorescein succinimidyl ester (CFSE) according to the manufacturer's instructions. Labeled HCC cells were seeded at a density of 5 × 10^4^ cells per well. NK92MI cells were then added to the wells at varying E:T ratios. The coculture was maintained at 37 °C in a humidified incubator with 5% CO_2_ for 8–24 h, depending on the experimental design. All of cells were stained with propidium iodide (PI) to identify dead cells after incubation. The samples were analyzed by flow cytometry. CFSE‐positive HCC cells and PI‐positive cells were quantified to determine the percentage of tumor cell lysis presenting NK cell cytotoxicity. Cytotoxicity % = (experimental PI/CFSE positive cells − spontaneous PI/CFSE positive cells)/(total CFSE positive cells) × 100%.

### Human CircRNA Microarray

The Human circRNA chip (ArrayStar) contained more than 40 000 probes specific for human circRNAs splicing sites was used in this study. 3 HCC WT and 3 HCC NK‐R cell lines were analyzed on the circRNA chips. Exogenous RNAs developed by the External RNA Controls Consortium were used as controls. The sequencing and analysis procedures were performed by Aksomics Co., Ltd.

### Lentiviral Infection and SiRNA Transfection

HEK293 cells were transfected with 20 µg core plasmid (LentiCRSPRv2, pLVX, pLO5‐ciR, or PCDH), 10 µg psPAX2 packaging plasmid, and 10 µg pMD2.G envelope plasmid, for 48 h to obtain the lentivirus supernatant and further produce stable polyclonal populations (1 mL lentivirus supernatant/1 × 10^6^ HCC cells for 8 h). Among them, pLO5‐ciR (without GFP sequence) core plasmid was specialized circRNA overexpression vectors contained circRNA overexpression framework (such as Alu element, QKI, and other RBP binding sites). EcoRI and BamHI cleavage sites were incorporated in the center of the expression framework, allowing for the direct insertion of the target circRNA sequence. For siRNA transfection, LipofectAMINE 3000 (Invitrogen) was used with manufacturer's instructions accordingly. Briefly, in order to successfully knockdown target, 0.2 nmol specific siRNA and equal volume µL LipofectAMINE 3000 were needed and mixed thoroughly. Culture medium should be changed 8 h after transfection and knockdown efficiency could be detected 48 h later. Also, sgRNAs for CRISPR were generated by LentiCRSPRv2 manufacturer's instructions.

### Quantitative Real‐Time PCR

Total RNAs of cultured cells, tumor tissues, and adjacent nontumor liver tissues were obtained using Trizol reagent (Invitrogen). Complementary DNA was synthesized using Superscript III transcriptase (Invitrogen). RT‐qPCR was performed using a Bio‐Rad CFX96 system (Bio‐Rad) with SYBR Green to assess the expression levels of the targets of interest. The detecting primers for circRNAs were designed based on its head‐to‐tail junction.

### Cell Proliferation, Migration, and Invasion Assays

Proliferation of cells was determined with CCK‐8 kit according to the manufacturer's protocol at specific time points over a 72 h period. For transwell assay, HCC cells were plated with serum‐free medium into the upper chamber at 0.2–1 × 10^5^ cells per well and the bottom chamber of the apparatus was added culture medium with 10% FBS. After 24 h incubation at 37 °C, migrated cells attached to the lower surface of the membrane were fixed by 4% paraformaldehyde and stained with 0.1% crystal violet for observation. The real‐time xCELLigence cell analyzer RTCA (Roche Diagnostics) was used to measure cell migration over time for a 60 h period. For 3D invasion assay, HCC spheroids were first generated (diameter = 50 µm) in Nunclon Sphera 96U‐well plate (Invitrogen) according to the manufacturer's instructions. After that, HCC spheroids were transferred to a medium containing matrix and invasion of spheroids in the matrix could be observed one week later.

### Flow Cytometry

NK92MI cells were stained with fluorochrome‐conjugated antibodies. Intracellular staining for cytokines was performed using the BD Cytofix/Cytoperm Plus Fixation/Permeabilization Solution Kit (BD Biosciences) and data were analyzed by FlowJo software.

### Glycolysis Stress Test and Mitochondrial Stress Test

ECAR and OCR were determined using the XF96 Extracellular Flux Analyzer (Seahorse Bioscience). Targeted cells were plated at a density of 1.0 × 10^4^ cells per well in an XF96 plate and incubated overnight. The media were exchanged to XF media 1 h before the assay. The XF Glycolysis Stress Test Kit and the XF Mito Stress Test Kit were employed to assess ECAR and OCR. For glycolysis stress test, glucose, oligomycin, and 2‐DG were diluted in XF media to achieve final concentrations of 10 mm, 1 µm, and 50 mm, respectively, and then loaded into the cartridges. For mitochondrial stress test, oligomycin, FCCP, and rotenone/antimycin A were diluted in XF media to achieve final concentrations of 1.5, 1, and 0.5 µm, respectively, and then loaded into the cartridges. ECAR and OCR measurements were conducted according to the manufacturer's instructions.

### Metabolite Extraction and Targeted Metabolomics Analysis

Collected HCC cell samples were diluted 100‐fold in water and thoroughly vortexed. Subsequently, 100 µL of these samples were homogenized with 500 µL of methanol/water (8:2) containing mixed internal standards, followed by vortexing. The mixture was then placed on ice for 30 min and centrifuged at 12 000 rpm for 10 min. The supernatant was finally injected into the LC–MS/MS system for targeted metabolomics central carbon metabolism analysis by Novegene Co., Ltd.

### Measurements of Lactate and Pyruvate

Lactate concentrations in supernatants of cells cultured for 24 h were determined using the l‐Lactic Acid Colorimetric Assay Kit (Elabscience) according to the manufacturer's protocol. Intracellular pyruvate levels were determined using the Pyruvic Acid Colorimetric Assay Kit (Elabscience) according to the manufacturer's protocol.

### RNA Pull‐Down and Purification

For circRNA pull‐down assays, cell lysates were prepared using IP lysis buffer and followed the manufacturer's instruction of Pierce Magnetic RNA–Protein Pull‐Down Kit (Thermo Fish Scientific). Briefly, washed Pierce Streptavidin Magnetic Beads were incubated with cell lysate at 4 °C for 1 h for preclearance. A 3′ biotin‐labeled circRNA probe was then added to the beads and incubated at room temperature for 10 min for immobilization. Subsequently, the biotinylated beads were incubated with cell lysate at 4 °C overnight, magnetically separated, and washed 3–5 times. For immunoblotting detection, the beads were boiled in sodium dodecyl sulfate (SDS) buffer for protein/peptide isolation. For mass spectrometry assays, the beads were incubated with nonionic water at 70 °C for 5 min. For RT‐qPCR assays and circRNA purification, Trizol reagent was used to extract total RNA from the beads.

### Mass Spectrometry Analysis

Peptide samples collected from HepG2‐R cells were analyzed on Q‐Extractive mass spectrometer (Thermo Fish Scientific) by Novegene Co., Ltd. The full mass and subsequent MS/MS analyses were conducted in an Orbitrap analyzer with a resolution of 70 000 for MS1 (at 200 mass/charge ratio [*m*/*z*]) and 17 500 for MS2, respectively. Automatic gain control target for MS1 was set to 3.0 × 10 + 6 with max inject time (IT) of 50 ms and 5.0 × 10 + 4 for MS2 with max IT 100 ms. The top 20 most intense ions were fragmented by HCD with a normalized collision energy of 27% and an isolation window of 2 *m*/*z*. Dynamic exclusion was set to 30 s. Raw data were processed using MaxQuant software (version 1.5.6.0). Intracellular pathway analysis was performed using the clusterProfiler in R package to search the Gene Ontology (GO) and Kyoto Encyclopedia of Genes and Genomes (KEGG) databases.

### Protein Extraction, Immunoprecipitation, and Immunoblotting

Cells were lysed in RIPA or IP lysis buffer supplemented with Protease Inhibitor Cocktail, Phosphatase Inhibitor Cocktail, Panobinostat, and Methylstat. Lysates were cleared by centrifugation (12 000 rpm, 15 min) at 4 °C. Supernatants were analyzed for immunoblotting or immunoprecipitated with the indicated antibodies. For immunoprecipitation, prepared cell lysates were precleared using Protein A+G Agarose beads by rotating at 4 °C for 1 h. The specified antibody and control IgG were added to the precleared lysates, followed by the addition of Protein A+G Agarose beads to the mixture. The tubes were incubated at 4 °C with gentle rotation for 3–5 h for each step. The protein‐captured beads were washed with lysis buffer 3–5 times and eluted with 50 µL SDS loading buffer for detection by immunoblot. Proteins were separated using 10–15% SDS/polyacrylamide gel electrophoresis gel and subsequently transferred onto PVDF membranes (Millipore). After blocking, the membranes were incubated with appropriate dilutions of specific primary antibodies, followed by incubation with HRP‐conjugated secondary antibodies. Protein detection was performed using the ECL system (Bio‐Rad).

### RNA FISH Assay and Immunofluorescence Staining

RNA FISH was conducted using a FISH kit (Ribobio) according to the manufacturer's instructions. Briefly, cells on chamber slides were fixed in 4% formaldehyde for 15 min. The air‐dried cells were then incubated with Cy3‐conjugated FISH probe (Ribobio) in hybridization buffer (100 mg mL^−1^ dextran sulfate, 10% formamide in 2× SSC) at 80 °C for 2 min. Hybridization was performed at 55 °C for 2 h, followed by washing the slides with 0.1× SSC at 65 °C and dehydrating through 70%, 90%, and 100% ethanol. The slides were subsequently used for further immunofluorescence staining or mounted with Prolong Gold Antifade Reagent containing DAPI for confocal microscopy detection. For immunofluorescence, cells were cultured overnight in chamber slides and fixed with 4% formaldehyde in phosphate‐buffered saline (PBS) for 15 min at 4 °C, followed by permeabilization with 0.5% Triton X‐100 in PBS for 15 min. After blocking nonspecific binding with 10% goat serum in PBS and 0.1% Tween‐20 overnight, cells were incubated with the specified antibody for 1 h at room temperature, followed by incubation with Goat anti‐Mouse or anti‐Rabbit IgG (H+L) Antibody from Abcam for 1 h. Immunofluorescence images were captured using an Olympus FV3000 fluorescence microscope, with consistent settings across all channels.

### RNA Immunoprecipitation and RNA Extraction

To prepare antibody‐coated beads, 20 µL of Protein A+G Agarose beads were incubated with 1–5 mg of indicated antibody or control IgG in 500 µL of lysis buffer supplemented with Protease Inhibitor Cocktail, Phosphatase Inhibitor Cocktail, Panobinostat, and Methylstat at 4 °C overnight. The beads were then washed twice with lysis buffer and stored on ice. Cell lysates were precleared using 5 µL of Protein A+G Agarose beads by rotation at 4 °C for 1 h. Precleared lysates were transferred to tubes containing the antibody‐coated beads and rotated at 4 °C for 3–5 h. The protein‐captured beads were washed 3–5 times with lysis buffer. RNA extraction from the beads was performed using Trizol according to the manufacturer's instructions for subsequent detection. Additionally, nuclear and cytoplasmic RNA isolation was carried out using the PARIS Kit (Thermo Fisher Scientific) following the manufacturer's protocol.

### CircRNA Structure Simulation and Analysis

UNAFold (version2.3, https://www.unafold.org/mfold/applications/rna‐folding‐form‐v2.php) was used to determine the circRNA secondary structures with minimum free energy. UNAFold employed energy minimization to identify the optimal folding of a nucleic acid sequence within a specified energy increment, thereby simulating the ensemble of possible structures. According to UNAFold calculations, the most favorable secondary structure for circSMPD4 had a free energy of −203.40 kcal mol^−1^. The secondary structure with the lowest theoretical free energy value was selected for subsequent 3D modeling. 3dRNA (http://biophy.hust.edu.cn/new/3dRNA/create/) was used to generate RNA 3D model. 3dRNA was a web‐based software for RNA folding and 3D structure prediction. 3dRNA was used to analyze the tertiary structure and generate the 3D model of circSMPD4.

### CircRNA/Protein Interaction Simulation and Analysis

The 3D model of circSMPD4 was put in HDOCK software (http://hdock.phys.hust.edu.cn/) together with LDHA (PDB No. AF‐P00338‐F1) for RNA/protein interaction simulation. Distance‐based approach was used to identify the binding site nucleotides/residues for the RNA–protein complexes using a specific cutoff value. Two atoms, one in RNA and another in protein, were considered to interact with each other if the distance between them was <4.5Å.

### Chemical Treatment and Inhibitor Incubation Assays

All the chemicals and inhibitors were prepared according to manufacturer's suggestion. Actinmyocin D was used in RNA decay assays at a concentration of 2 µg mL^−1^. Glycolysis inhibitor 2‐DG was used at the concentration of 10 mm, lactate was used at the concentration of 2–10 mm as indicated in specific assays. CHX was used in LDHA degradation assay at a concentration of 5 µg mL^−1^. UAE inhibitors PYR‐41 and TAK‐243 treated cells at the concentration of 20 and 1 µm, respectively. Proteasome inhibitor MG‐132 treated cells at the concentration of 20 µm. Lysosomal inhibitors CQ and leupeptin treated cells at the concentration of 20 and 50 µm, respectively. Deacetylase inhibitors TSA and NAM treated cells at the concentration of 5 nm and 20 mm, respectively.

### Quantification and Statistical Analysis

GraphPad Prism v.9.5.1 was used to calculate statistical significance. Experiments were set up to use at least 3 samples (biological replicates). Results were reported as mean ± standard deviation (SD). Comparisons were performed using paired/no‐paired Student's *t*‐test or two‐way ANOVA test, as indicated in individual figures. Kaplan–Meier survival curves were compared using the Gehan–Breslow test in GraphPad Prism v9.5.1.

## Conflict of Interest

The authors declare no conflict of interest.

## Author Contributions

H.S., B.L., and J.H. contributed equally to this work. H.S., X.C., L.S., and Y.W. designed the study. H.S. performed most of the biochemical and molecular experiments, with the assistance from B.L., J.H., W.Z., W.L., L.M., and L.H. J.H. performed bioinformatics analysis and interpreted the data. B.L. and W.Z. collected and provided clinical samples and data. H.S. and B.L. wrote the paper, L.S. and Y.W. revised the paper. All of the authors read and commented on the paper.

## Supporting information



Supporting Information

## Data Availability

The data supporting the findings of this study are available on reasonable request from the lead contact, Liang Shi (liang_shi@zju.edu.cn).
