# Supplementary material for: Lactate Metabolic Reprogramming Mediated by CircRNA–LDHA Complex Facilitates Innate Immune Evasion of Liver Cancer
Source: Adv Sci (Weinh). 2025 Sep 12;12(45):e09989. doi: 10.1002/advs.202509989 (PMC12677679; doi:10.1002/advs.202509989)
Supplement: Supplementary file 1 — Supporting Information [file ADVS-12-e09989-s001.docx]

**Supporting Information**

**Lactate Metabolic Reprogramming Mediated by circRNA-LDHA Complex Facilitates Innate Immune Evasion of Liver Cancer**

Hao Shen, Boqiang Liu, Jing He, Weijun Zhao, Weiqi Li, Lingfeng Ma, Lidan Hou, Yi Wang, Chenqi Jin, Yushun Chang, Jie Lin, Jia Zhao, Binghan Jin, Yuanshi Tian, Xiujun Cai*, Liang Shi*, Yifan Wang*

**
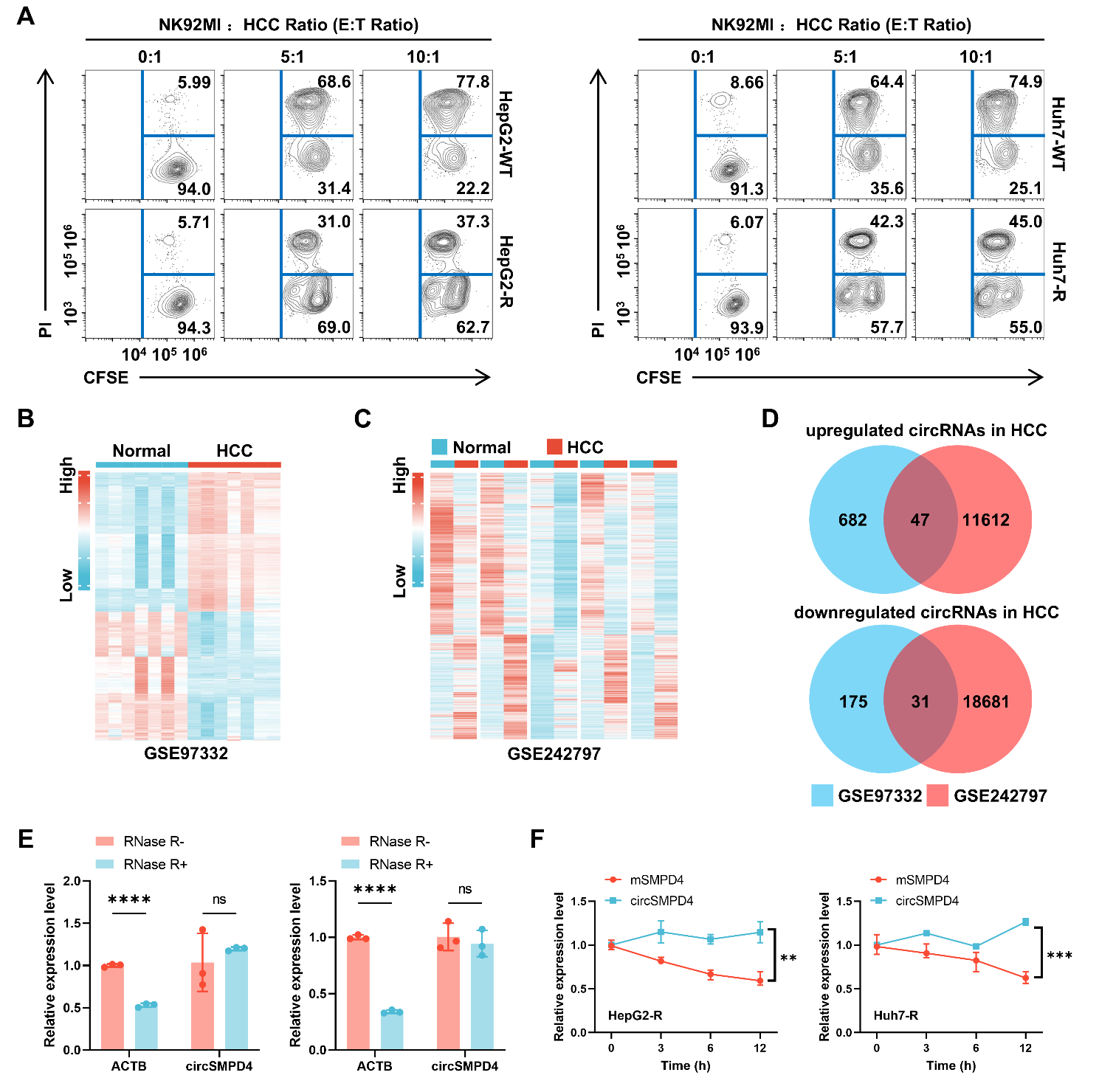
**

**Figure S1. Identification of circSMPD4 as an Immunosuppressive and Oncogenic circRNA in NK Cell-Drived Tumor Evolution. (A)** Representative pictures of NK cell cytotoxicity assays on 2 groups (HepG2 and Huh7) of WT & NK-R cell lines using flow cytometry, NK92MI cells were added at different E:T ratio. **(B, C)** The Heatmap of differentially expressed circRNAs between normal tissues/non-tumor tissues and HCC tissues in GSE97332 (B) and GSE242797 (C). **(D)** A Venn Diagram showing differentially expressed circRNAs within GSE97332 and GSE242797. **(E)** RT-qPCR analysis of circSMPD4 after RNase R treatment in HepG2-R (left) and Huh7-R (right) cells (n=3 independent biological replicates per group). ACTB mRNA was a negative control (mean ± SD, ****P < 0.0001, n.s., not significant, unpaired Student’s t-test). **(F)** The relative RNA levels were examined by RT-qPCR after treating with actinomycin D at the indicated time points in HepG2-R (left) and Huh7-R (right) cells, n=3 independent biological replicates per group (mean ± SD, **P< 0.01, ***P < 0.001, two-way ANOVA test).

**
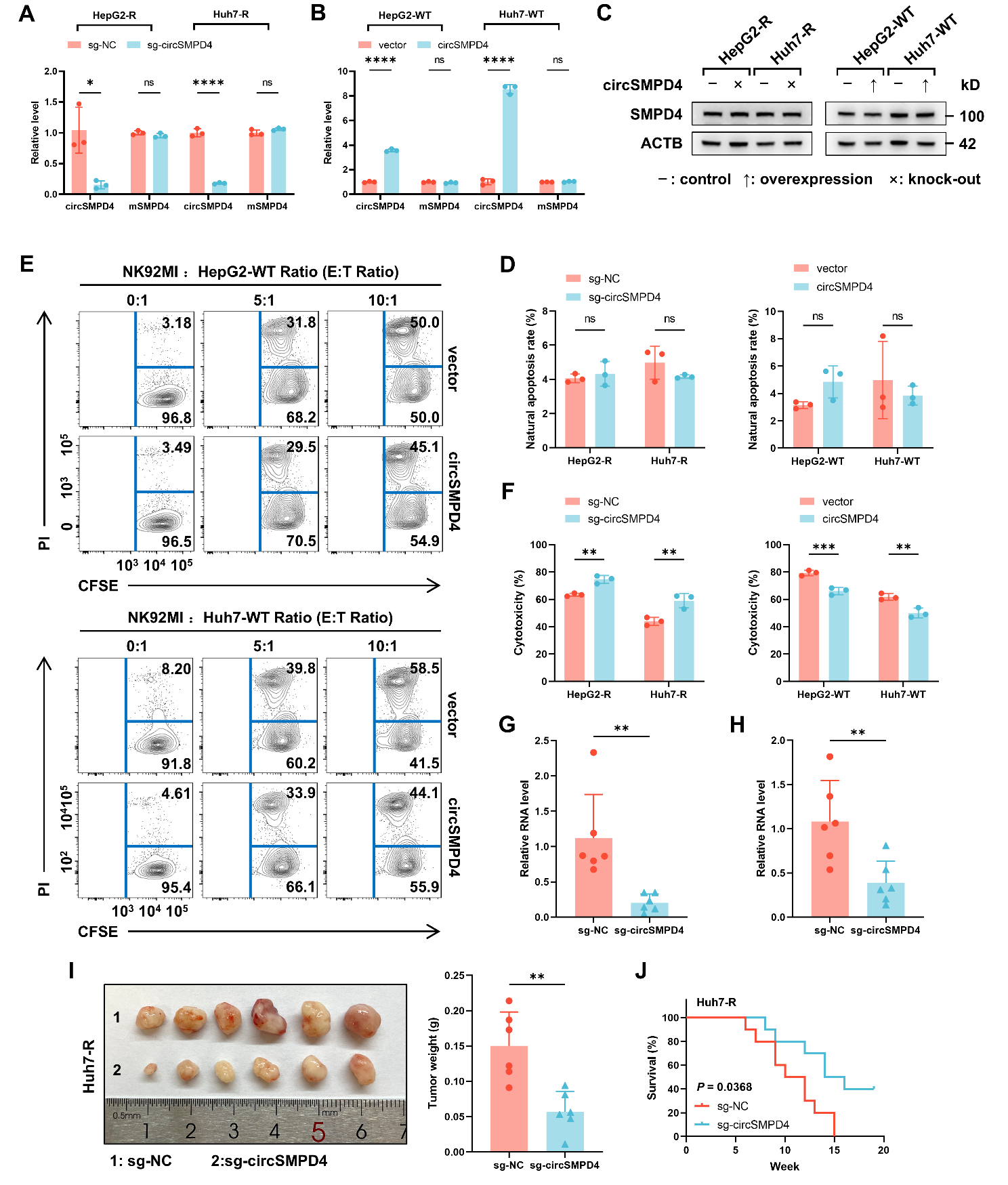
**

**Figure S2.** **circSMPD4 Mediates Resistance to NK Cell Cytotoxicity and Enhances Tumor Malignancy. (A, B)** Confirmation of circSMPD4 knockout (A) in NK-R cells or overexpression (B) in WT cells by RT-qPCR, n=3 independent biological replicates per group (*P < 0.05, ****P < 0.0001, n.s., not significant, unpaired Student’s t-test). ACTB was used as an internal reference. **(C)** Tumor cells with circSMPD4 knockout or overexpression were analyzed for SMPD4 expression. ACTB was used as an internal reference. **(D)** Quantification of the natural apoptosis rate of various tumor cells at an E:T ratio of 0:1 using flow cytometry, n=3 independent biological replicates per group (n.s., not significant, unpaired Student’s t-test). **(E)** Representative pictures of NK cell cytotoxicity assays on 2 pairs WT & NK-R cell lines with circSMPD4 overexpression using flow cytometry, NK92MI cells were added at different E:T ratio. **(F)** Quantification of NK cell cytotoxicity in fluorescent-labeled NK cell cytotoxicity assays, n=3 independent biological replicates per group (**P < 0.01, ***P < 0.001, unpaired Student’s t-test). **(G, H)** Confirmation of circSMPD4 knockout in the orthotopic tumors (F, HepG2-R and G, Huh7-R) by RT-qPCR, n=6 mice per group (**P < 0.01, unpaired Student’s t test). ACTB was used as an internal reference. **(I)** Orthotopic tumors of indicated Huh7-R cells were isolated from mouse liver (left) and the weight of each tumor was quantified (right), n=6 mice per group (mean ± SD. *P < 0.05, unpaired Student’s t-test). **(J)** Kaplan-Meier curves showing overall survival of 20 mice after orthotopic tumor transplantation with indicated Huh7-R tumors followed up to 18 weeks, n=10 mice per group (*P < 0.01, Gehan-Breslow test).

**
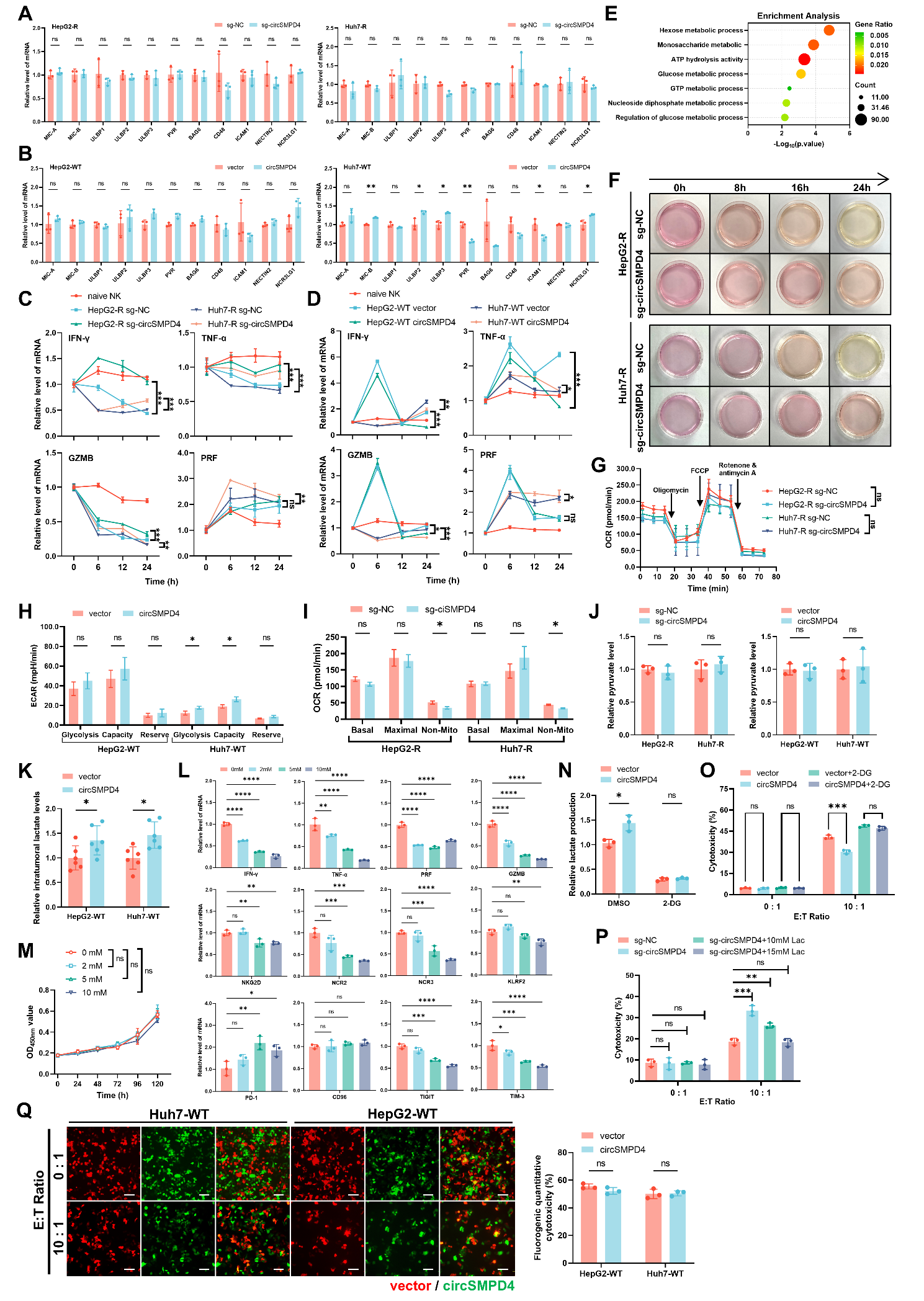
**

**Figure S3. circSMPD4 Impairs NK Cell Function by Enhancing the Warburg Effect of HCC. (A, B)** Expression levels of NK activating ligands in tumor cell lines with circSMPD4 knockout (A) or overexpression (B) were quantified using RT-qPCR, n=3 independent biological replicates per group (*P < 0.05, **P < 0.01, n.s., not significant, unpaired Student’s t-test). **(C, D)** Direct co-culture experiments between NK cells and tumor cells with circSMPD4 knockout (C) or overexpression (D). NK92MI cells were collected at specific time points and the expression levels of activating effector molecules were assessed using RT-qPCR, n=3 independent biological replicates per group (means ± SD, *P < 0.05, **P < 0.01, ***P < 0.001, n.s., not significant, two-way ANOVA test). **(E)** Metabolism-related pathways exhibiting significant differences in enrichment analysis of transcriptome sequencing on 3 WT and 3 NK-R cell lines. **(F)** Plate equal amounts of tumor cells, equivalent to the maximum cell capacity of the culture dish. After cells adhere, images of the medium color at indicated time points were captured. Representative images are shown. **(G)** Oxygen Consumption rate of tumor cells with circSMPD4 knockout (mean ± SD, n.s., not significant, two-way ANOVA test, n= 3 independent samples per group). **(H)** Glycolysis, Glycolytic capacity and Glycolytic reserve levels were quantified from glycolysis stress test assays of tumor cells with circSMPD4 overexpression, n= 4 independent samples per group (mean ± SD, *P < 0.05, n.s., not significant, unpaired Student’s t-test). **(I)** Basal, maximal and non-mitochondrial respiration levels were quantified from mitochondrial stress test assays of tumor cells with circSMPD4 knockout, n= 4 independent samples per group (mean ± SD, *P < 0.05, n.s., not significant, unpaired Student’s t-test). **(J)** Measurement of intracellular levels of pyruvate in tumor cell lines with circSMPD4 knockout or overexpression, n= 3 independent samples per group (mean ± SD, n.s., not significant, unpaired Student’s t-test). **(K)** Measurement of intratumoral lactate levels in indicated HepG2-WT or Huh7-WT tumors, n=6 mice per group (mean ± SD, *P < 0.05, unpaired Student’s t-test). **(L)** Expression levels of cytokine/cytotoxin (IFN-γ, TNF-α, PRF, GZMB), activating/recognition receptors (NKG2D, NCR2, NCR3, KLRF2) or inhibitory receptors (PD-1, CD96, TIGIT, TIM-3) of NK92MI cells treated with indicated lactate concentration were assessed using RT-qPCR, n=3 independent biological replicates per group (mean ± SD, *P < 0.05, **P < 0.01, ***P < 0.001, ****P < 0.0001, n.s., not significant, unpaired Student’s t-test). **(M)** CCK-8 assays of NK92MI cells treated with indicated lactate concentration, OD450nm value were detected every 24h, n=3 independent biological replicates per group (n.s., not significant, two-way ANOVA test). **(N)** Measurement of supernatant levels of lactate in Huh7-WT cell lines with circSMPD4 overexpression treated with 2-DG, n=3 independent biological replicates per group (mean ± SD, *P < 0.05, n.s., not significant, unpaired Student’s t-test). **(O)** Huh7-WT cells with circSMPD4 overexpression were subjected to NK cell cytotoxicity assays after treating with 2-DG at various E:T ratios using flow cytometry, cytotoxicity was quantified to perform in histogram, n=3 independent biological replicates per group (mean ± SD, ***P < 0.001, n.s., not significant, unpaired Student’s t-test). **(P)** HepG2-R cells with circSMPD4 knockout were subjected to NK cell cytotoxicity assays after treating with indicated lactate concentration at various E:T ratios using flow cytometry, cytotoxicity was quantified to perform in histogram, n=3 independent biological replicates per group (mean ± SD, **P < 0.01, ***P < 0.001, n.s., not significant, unpaired Student’s t-test). **(Q)** Tumor normal control cells labeled with RFP and circSMPD4 overexpression cells labeled with GFP were mixed in a 1:1 ratio and plated. NK92MI cells were added in different E:T ratios and co-cultured for 24 hours. After removing NK cells and dead cells, fluorescence images were captured. Left: Representative fluorescence images were shown. Scale bar, 100 μm. Right: Quantification of NK cell cytotoxicity, n=3 independent biological replicates per group (n.s., not significant, unpaired Student’s t-test).

**
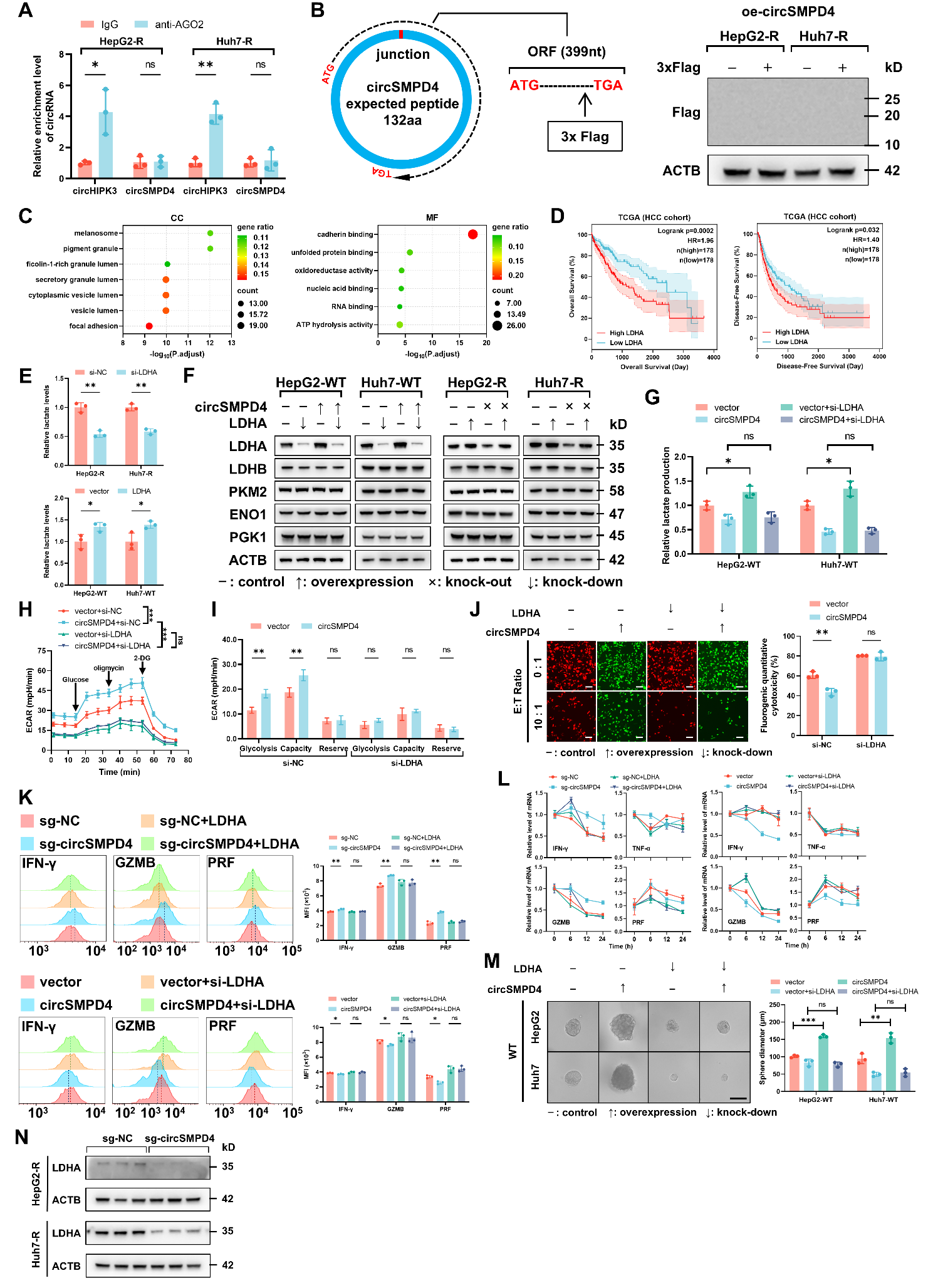
**

**Figure S4. The circSMPD4/LDHA Axis Mediates circSMPD4's Biological Functions. (A)** AGO2 RIP assays showed circSMPD4 cannot enriched by AGO2, while circHIPK3 (a well-known circRNA functioned as miRNA sponge) was used as a positive control, n=3 independent biological replicates per group (*P < 0.05, **P < 0.01, and n.s., not significant, unpaired Student’s t-test). **(B)** Left: ORF-Flag (cross junction-site) fusion construct was transfected into NK-R cells. The length of expected peptide encoded by circSMPD4 is 132aa (about 10-15 kD). Right: 132aa-Flag fusion protein was detected by immunoblotting. ACTB was used as an internal reference. **(C)** Molecular function (MF) and cellular component (CC) in gene ontology (GO) analysis of circSMPD4-interacting proteins. **(D)** Kaplan–Meier curves showing overall survival (left) and disease-free survival (right) of HCC patients from TCGA cohort. Patients were separated by the expression level of LDHA, Gehan-Breslow test. **(E)** Measurement of supernatant levels of lactate in tumor cell lines with LDHA knockdown or overexpression, n=3 independent biological replicates per group (mean ± SD, *P < 0.05, **P < 0.01, unpaired Student’s t-test). **(F)** Confirmation of LDHA knockdown or overexpression in various tumor cell lines by immunoblotting. Other related metabolic enzymes were used as negative controls. ACTB was used as an internal reference. **(G)** Knockdown of LDHA revoked the function of circSMPD4 on lactate production (*P < 0.05, n.s., not significant, unpaired Student’s t-test). **(H, I)** Knockdown of LDHA eliminated circSMPD4-mediated Warburg effect on extracellular acidification rate (H, ***P < 0.001, n.s., not significant, two-way ANOVA test) and glycolysis ability (I, **P < 0.01, n.s., not significant, unpaired Student’s t-test), n=4 independent biological replicates per group. **(J)** Fluorescent-labeled NK cell cytotoxicity assays in indicated groups with or without LDHA knockdown. Left: Representative fluorescence images were shown. Scale bar, 100 μm. Right: Quantification of NK cell cytotoxicity, n=3 independent biological replicates per group, **P < 0.01, n.s., not significant, unpaired Student’s t-test). **(K, L)** Indirect co-culture experiments between NK cells and tumor cells in indicated groups, n=3 independent biological replicates per group. The expression levels of NK cell effector molecules were quantified by flow cytometry (K. Left: Representative images were shown. Right: Fluorescence intensity was quantified, *P < 0.05, **P < 0.01, n.s., not significant, unpaired Student’s t-test) or assessed using RT-qPCR (L). **(M)** *In vitro* 3D invasion assays were performed in indicated groups. Left: Representative pictures were shown. Right: The diameter of tumor sphere was measured and analyzed, n=3 independent biological replicates per group (**P < 0.01, ***P < 0.001, unpaired Student’s t-test). Scale bar, 100 μm. **(N)** Immunoblot analysis from orthotopic tumor models established using indicated cells in NCG mice. ACTB was used as an internal reference.

**
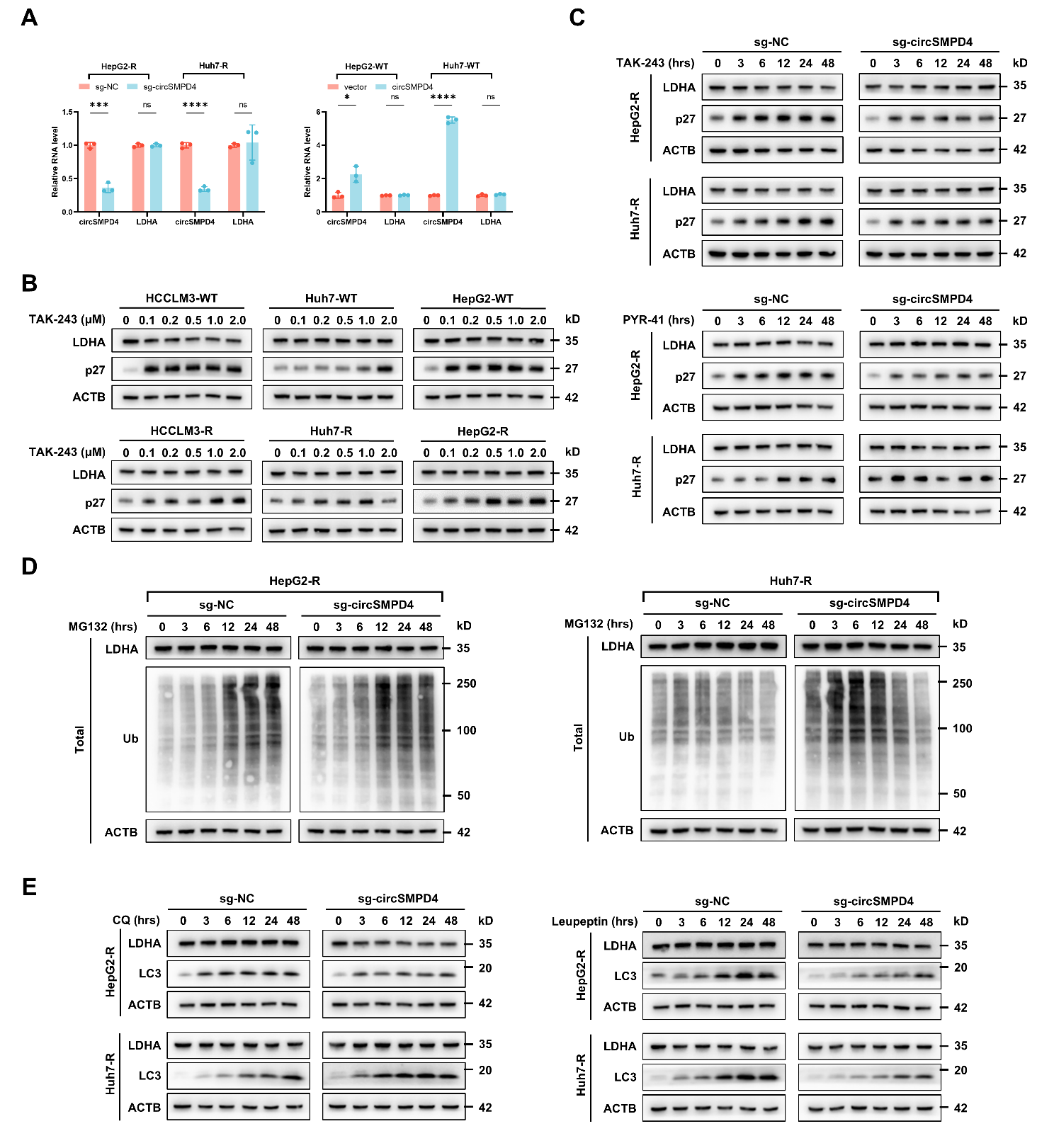
**

**Figure S5.** **circSMPD4 Inhibits the CMA-Mediated Degradation of LDHA. (A)** Transcriptional levels of LDHA in tumor cells ± circSMPD4 knockout or overexpression were determined by RT-qPCR, n=3 independent biological replicates per group (*P < 0.05, ***P < 0.001, ****P < 0.0001, n.s., not significant, unpaired Student’s t-test). **(B)** 3 pairs (HepG2, Huh7, HCCLM3) of WT & NK-R cells were treated with TAK-243 at indicated concentration for 24h. Endogenous level of LDHA was determined by immunoblotting. P27 was used as a positive control. **(C)** Tumor cells ± circSMPD4 knockout were treated with TAK-243 (1 μM) or PYR-41 (20 μM) and LDHA level was determined at indicated time points by immunoblotting. P27 was used as a positive control. **(D)** Tumor cells ± circSMPD4 knockout were treated with MG-132 (20 μM). LDHA and Ubiquitin (Ub) levels were determined at indicated time points by immunoblotting. **(E)** Tumor cells ± circSMPD4 knockout were treated with CQ (20 μM) or Leupeptin (50 μM). LDHA and LC3 levels were determined at indicated time points by immunoblotting.

**
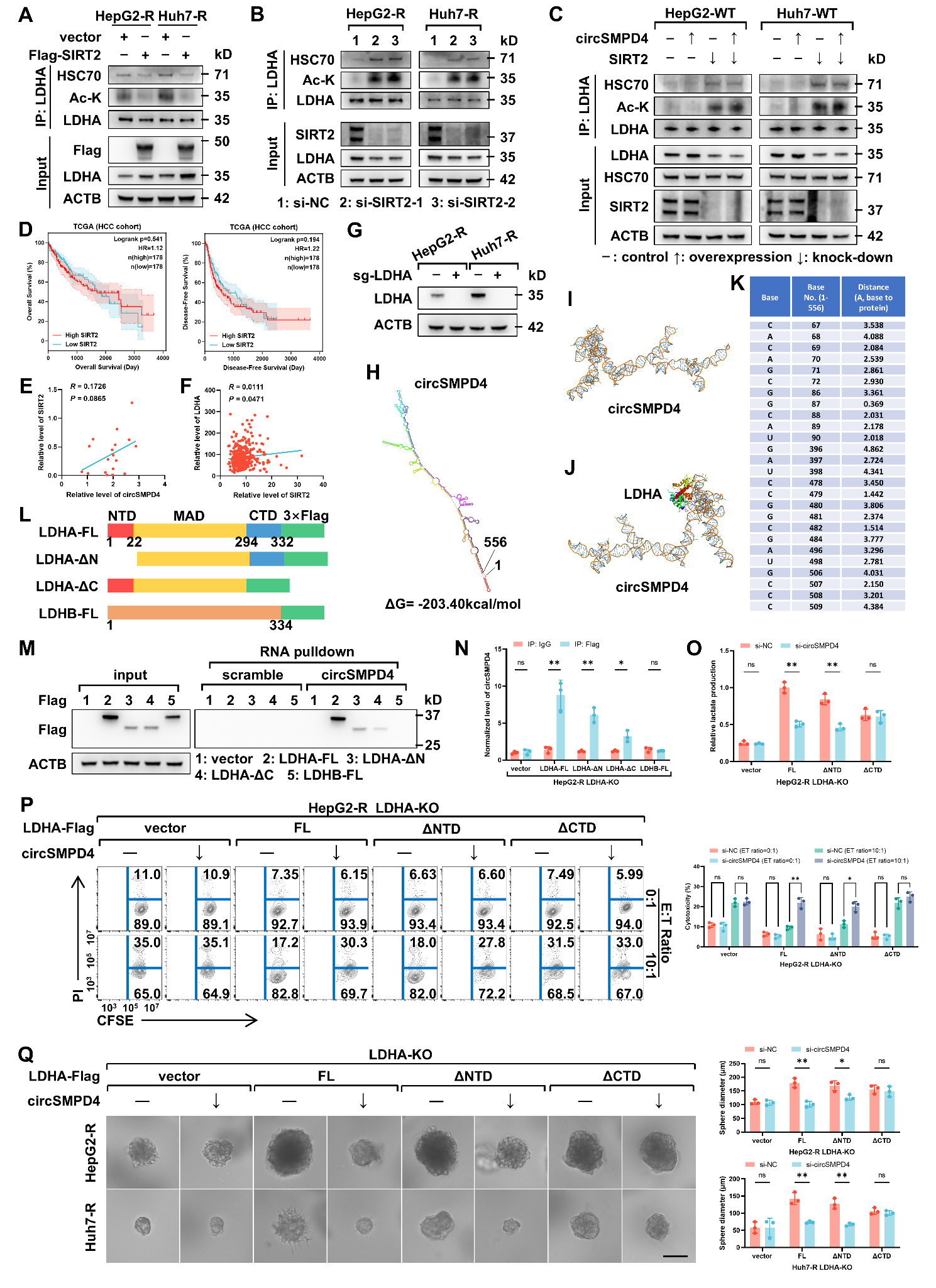
**

**Figure S6. circSMPD4 Promotes SIRT2-Mediated Deacetylation of LDHA and Its Interaction with C-terminus of LDHA is Vital for Biological Function. (A, B)** Overexpression or knockdown of SIRT2 was revealed to affect the acetylation level of LDHA and the combination between LDHA and HSC70 in tumor cells. LDHA acetylation and protein levels were determined by immunoblotting with indicated antibody. **(C)** Knockdown of SIRT2 remarkably blocked circSMPD4-mediated LDHA upregulation and deacetylation, meanwhile facilitating the combination between LDHA and HSC70. LDHA acetylation, protein levels of LDHA, HSC70 and SIRT2 were determined by immunoblotting. **(D)** Kaplan–Meier curves showing overall survival (left) and disease-free survival (right) of HCC patients from TCGA cohort. Patients were separated by the expression level of SIRT2, Gehan-Breslow test. **(E)** Correlations between expression levels of circSMPD4 and SIRT2 in HCC tissues (cohort 1). RNA levels were determined using RT- qPCR and normalized to ACTB. The R values and P values were calculated using Pearson correlation analysis. **(F)** Correlations between expression levels of SIRT2 and LDHA in HCC tissues from TCGA cohort. The R values and P values were calculated using Pearson correlation analysis. **(G)** Confirmation of LDHA-KO in HepG2-R and Huh7-R cells. LDHA protein level was determined by immunoblotting. **(H)** Predicted circSMPD4 secondary structure with the lowest free energy made by Mfold 2.3 software. **(I)** A three-dimensional structure model of circSMPD4 generated by 3dRNA. **(J)** Simulated interaction between circSMPD4 and LDHA generated by HDOCK. **(K)** A diagram showing circSMPD4 bases predicted to interact with LDHA. **(L)** Image depicting the structure and sequence of LDHA-3×Flag. **(M)** LDHA-KO HepG2-R cells were transduced with LDHA^FL^-3×Flag, LDHA^ΔNTD^-3×Flag, LDHA^ΔCTD^-3×Flag, and LDHB^FL^-3×Flag, respectively. The level of LDHA-3×Flag enriched by circSMPD4-pulldown was determined by immunoblotting. **(N)** RNA immunoprecipitation assays with endogenous circSMPD4 as determined by RT-qPCR in indicated cells, n=3 independent biological replicates per group (mean ± SD, *P < 0.05, **P < 0.01, n.s., not significant, unpaired Student’s t-test). **(O)** Measurement of supernatant levels of lactate in indicated cells, n=3 independent biological replicates per group. LDHA-KO HepG2-R cells were reconstituted with LDHA^FL^, LDHA^ΔNTD^ and LDHA^ΔCTD^, respectively (mean ± SD, **P < 0.01, n.s., not significant, unpaired Student’s t-test). **(P)** LDHA-KO HepG2-R cells were reconstituted with LDHA^FL^, LDHA^ΔNTD^ and LDHA^ΔCTD^, respectively. Indicated cells were subjected to NK cell cytotoxicity assay at various E:T ratios using flow cytometry. Representative images were shown (Left) and results were quantified, n=3 independent biological replicates per group (Right, *P < 0.05, **P < 0.01, n.s., not significant, unpaired Student’s t-test). **(Q)** LDHA-KO HepG2-R and LDHA-KO Huh7-R cells were reconstituted with LDHA^FL^, LDHA^ΔNTD^ and LDHA^ΔCTD^, respectively. Indicated cells were subjected to *in vitro* 3D invasion assay. Representative images were shown (Left) and the diameter of tumor sphere was measured and analyzed, n=3 independent biological replicates per group (Right, *P < 0.05, **P < 0.01, n.s., not significant, unpaired Student’s t-test). Scale bar, 100 μm.

**
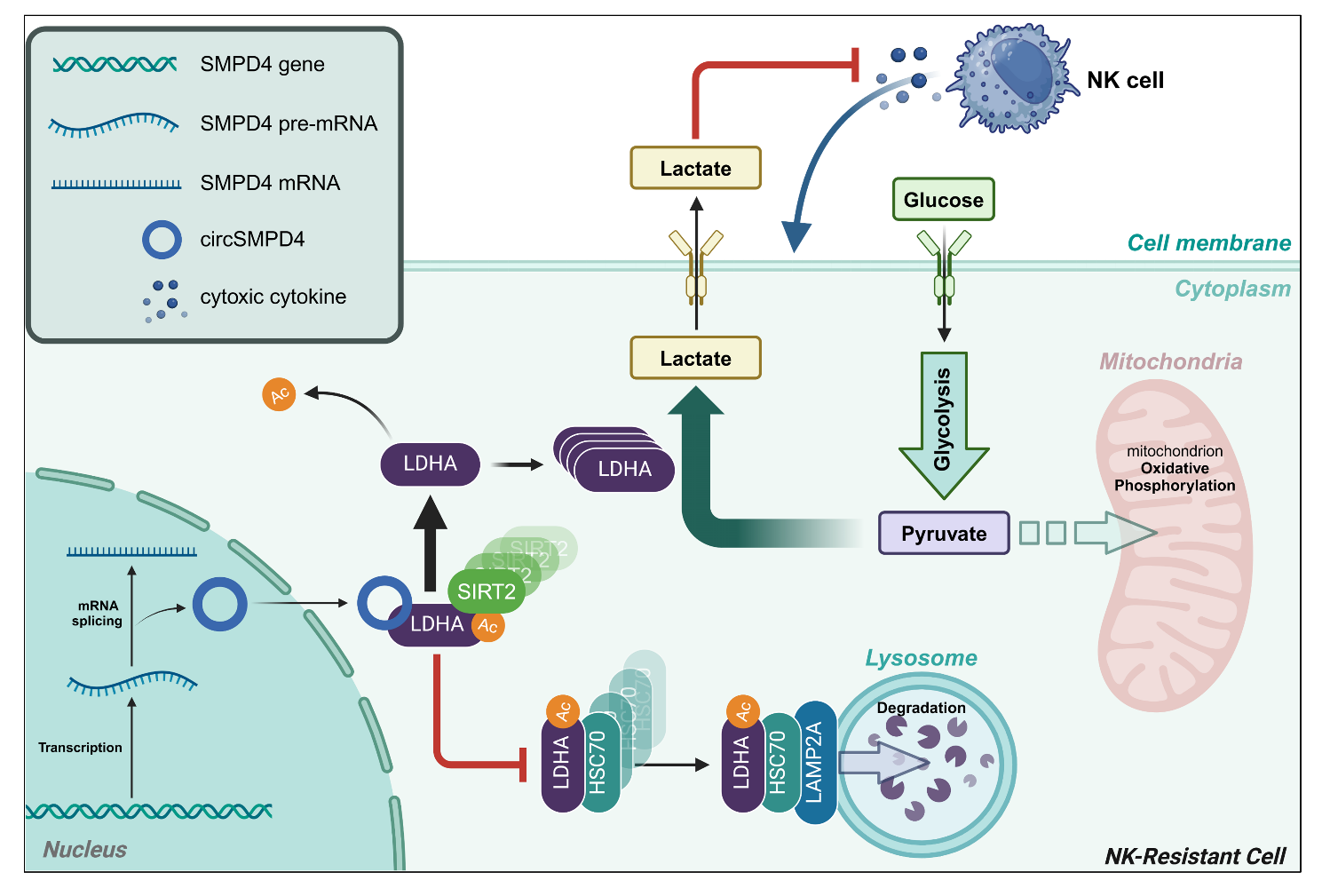
**

**Figure S7. Schematic diagram**

circSMPD4 physically combines with LDHA and reduces its acetylation level via SIRT2-dependent deacetylation, thereby inhibiting CMA-lysosome degradation of LDHA. Upregulating LDHA leads to increasing lactate production and extracellular export, which in turn suppresses NK cell cytotoxicity and induces immune evasion, while simultaneously promoting HCC development. This figure was created with BioRender.com.

**Supplemental Tables**

**Table S1. Clinicopathological analyses of 40 primary HCC patients with different expression levels of circSMPD4 (cohort 4)**

| Variables | | circSMPD4 | | *P* value |
| --- | --- | --- | --- | --- |
|  |  | High | Low |  |
| Age, years | ≤60 | 12 | 11 | 0.749 |
|  | >60 | 8 | 9 |  |
| Gender | Female | 1 | 1 | 1.000 |
|  | Male | 19 | 19 |  |
| HBV | No | 0 | 1 | 0.311 |
|  | Yes | 20 | 19 |  |
| Cirrhosis | No | 12 | 13 | 0.744 |
|  | Yes | 8 | 7 |  |
| AFP, ng/mL | ≤400 | 12 | 17 | 0.077 |
|  | >400 | 8 | 3 |  |
| Tumor size, cm | ≤5 | 7 | 11 | 0.204 |
|  | >5 | 13 | 9 |  |
| Recurrence | No | 7 | 11 | 0.204 |
|  | Yes | 13 | 9 |  |

**Table S2. Predicted binding site nucleotides/residues in the highest score model of circSMPD4-LDHA complexes**

| AA - Base pair(s) | Distance  (Base to AA) |
| --- | --- |
| 13A - 397 | 2.724 |
| 13A - 398 | 4.341 |
| 14A - 396 | 4.862 |
| 14A - 397 | 3.692 |
| 20A - 89 | 3.026 |
| 20A - 90 | 2.108 |
| 21A - 88 | 4.071 |
| 21A - 89 | 2.178 |
| 21A - 90 | 3.506 |
| 42A - 479 | 3.201 |
| 43A - 478 | 4.85 |
| 43A - 479 | 4.282 |
| 90A - 88 | 3.176 |
| 90A - 89 | 4.013 |
| 130A - 87 | 3.955 |
| 130A - 88 | 2.962 |
| 131A - 88 | 4.748 |
| 132A - 87 | 4.684 |
| 132A - 88 | 3.09 |
| 171A - 481 | 4.432 |
| 171A - 482 | 4.191 |
| 181A - 496 | 3.296 |
| 181A - 498 | 2.781 |
| 183A - 481 | 2.374 |
| 183A - 482 | 3.277 |
| 183A - 498 | 3.647 |
| 184A - 481 | 4.094 |
| 184A - 484 | 3.777 |
| 186A - 67 | 3.538 |
| 186A - 481 | 3.778 |
| 186A - 482 | 1.514 |
| 188A - 68 | 4.927 |
| 188A - 482 | 4.752 |
| 202A - 68 | 4.777 |
| 203A - 68 | 4.51 |
| 205A - 67 | 4.489 |
| 207A - 67 | 4.68 |
| 207A - 484 | 4.195 |
| 208A - 67 | 4.298 |
| 208A - 68 | 4.747 |
| 261A - 479 | 2.634 |
| 261A - 480 | 3.846 |
| 263A - 88 | 4.805 |
| 264A - 88 | 4.086 |
| 264A - 89 | 3.038 |
| 265A - 478 | 3.45 |
| 265A - 479 | 1.442 |
| 265A - 506 | 4.983 |
| 266A - 87 | 4.049 |
| 266A - 88 | 4.389 |
| 266A - 506 | 4.031 |
| 266A - 507 | 2.15 |
| 266A - 508 | 4.507 |
| 267A - 479 | 4.303 |
| 267A - 480 | 3.924 |
| 268A - 507 | 3.354 |
| 268A - 508 | 3.329 |
| 269A - 480 | 3.806 |
| 269A - 481 | 4.339 |
| 270A - 482 | 3.746 |
| 297A - 86 | 4.896 |
| 297A - 87 | 0.369 |
| 297A - 88 | 2.031 |
| 297A - 506 | 4.821 |
| 297A - 507 | 3.112 |
| 297A - 508 | 3.591 |
| 298A - 86 | 3.361 |
| 298A - 87 | 2.189 |
| 298A - 88 | 4.65 |
| 298A - 508 | 3.201 |
| 298A - 509 | 4.384 |
| 301A - 508 | 4.307 |
| 302A - 72 | 4.663 |
| 303A - 71 | 4.423 |
| 304A - 69 | 4.407 |
| 304A - 70 | 2.915 |
| 305A - 69 | 4.534 |
| 305A - 70 | 2.539 |
| 305A - 71 | 2.861 |
| 305A - 72 | 2.93 |
| 306A - 68 | 4.088 |
| 306A - 69 | 4.984 |
| 306A - 70 | 4.583 |
| 307A - 68 | 4.666 |
| 307A - 69 | 2.084 |
| 307A - 70 | 3.123 |

**Supplementary Materials**

**Antibodies**

| Name | Supplier | Cat No. |
| --- | --- | --- |
| Rabbit anti-ENO1 antibody [EPR10863(B)] | Abcam | Cat# ab155102 |
| Rabbit anti-HSC70 antibody [EP1531Y] | Abcam | Cat# ab51052 |
| Rabbit anti-LAMP2A antibody [EPR4207(2)] | Abcam | Cat# ab125068 |
| Mouse anti-DDDDK tag (Binds to FLAG® tag sequence) antibody [M2] (HRP) | Abcam | Cat# ab49763 |
| Rabbit IgG, monoclonal [EPR25A] - Isotype Control | Abcam | Cat# 172730 |
| Mouse IgG, monoclonal - Isotype Control | Abcam | Cat# 37355 |
| Goat anti-Mouse IgG (H+L) Antibody, Alexa Fluor 488 | Invitrogen | Cat# A32723 |
| Goat anti-Rabbit IgG (H+L) Antibody, Alexa Fluor 555 | Invitrogen | Cat# A32732 |
| Goat anti-Rabbit IgG (H+L) Antibody, Alexa Fluor 647 | Invitrogen | Cat# A32733 |
| Rabbit anti-LDHA antibody (C4B5) | Cell Signaling Technology | Cat# 3582T |
| Rabbit anti-PKM2 antibody (D78A4) | Cell Signaling Technology | Cat# 4053T |
| Rabbit anti-PKM1 antibody (D30G6) | Cell Signaling Technology | Cat# 7067T |
| Rabbit anti- LC3B antibody (D11) | Cell Signaling Technology | Cat# 3868T |
| Rabbit anti-Acetylated-Lysine antibody | Cell Signaling Technology | Cat# 9441S |
| Mouse anti-Beta Actin antibody | Proteintech | Cat# 66009-1-Ig |
| Rabbit anti-LDHA antibody | Proteintech | Cat# 19987-1-AP |
| Rabbit anti-SMPD4 antibody | ABclonal | Cat# A15473 |
| Mouse anti-LDHA antibody (E-9) | Santa Cruz Biotechnology | Cat# sc-137243 |
| Mouse anti-LDHB antibody (431.1) | Santa Cruz Biotechnology | Cat# sc-100775 |
| Mouse anti-Ubiquitin antibody (P4D1) | Santa Cruz Biotechnology | Cat# sc-8017 |
| Rabbit anti-PGK1 antibody | Bioss | Cat# bsm-61216R |
| Rabbit anti-SIRT2 antibody | Bioss | Cat# bsm-52221R |
| Brilliant Violet 421 anti-IFNγ antibody | Univ Bio | Cat# 564791 |
| PerCP/Cyanine5.5 anti-Perforin antibody | Univ Bio | Cat# 563762 |
| PE-CY7 anti TNFα antibody | Univ Bio | Cat# 560923 |
| FITC anti-GZMB antibody | Univ Bio | Cat# 561998 |
| ANTI-FLAG® M2 Affinity Gel | Sigma-Aldrich | Cat# A2200 |

**Cell lines**

| Name | Supplier | Cat No. |
| --- | --- | --- |
| Human: NK92MI | Procell | Cat# CL-0533 |
| Human: LO2 | Institute for Advanced Study of Central South University | Cat# HTCC01 |
| Human: HepG2 | ATCC | Cat# HB-8065 |
| Human: Huh7 | JCRB | Cat# JCRB0403 |
| Human: HCCLM3 | Type Culture Collection of Chinese Academy of Science | Cat# SCSP-528 |
| Human: HEK293 | ATCC | Cat# CRL-1573 |

**Primer and oligo sequences**

| Name | Sequence | Supplier |
| --- | --- | --- |
| circSMPD4 biotinylated probe | aaaCCGCAAGGGCCACCCACTCACCTGCTGCTTGCCC | TsingKe |
| circSMPD4 antisense biotinylated probe | aaaGGCGTTCCCGGTGGGTGAGTGGACGACGAACGGG | TsingKe |
| circASH2L biotinylated probe | aaaTAAATGGTATAATATCCTCGATCATGTAGGG | TsingKe |
| circSMPD4 probe with Cy3 conjugated | aaaCCGCAAGGGCCACCCACTCACCTGCTGCTTGCCC | Ribobio |
| si-circSMPD4 | GCCACCCACTCACCTGCTG | Ribobio |
| si-mSMPD4 | CATGATAGGCTTGGATTTC | Ribobio |
| si-LDHA | GAGATGATGGATCTCCAACA | Ribobio |
| si-SIRT2 | GCCATCTTTGAGATCAGCTAT | TranSheepBio |
| sgRNA-circSMPD4 | GTGAGGGTTGGCTCCGCCG | TsingKe |
| sgRNA-LDHA | CAACTGTAATCTTATTCTGG | TsingKe |
| circSMPD4 qPCR primer-F | GGGCTGTTTGTATGGGACGG | TsingKe |
| circSMPD4 qPCR primer-R | CTGTGGGCGACACCTTAGGA | TsingKe |
| circSMPD4-Mut qPCR primer-F | AGAATGGGGCTGAACCTCC | TsingKe |
| circSMPD4-Mut qPCR primer-R | CGACACCTTAGGAGGGGAAAG | TsingKe |
| SMPD4 qPCR primer-F | CCACGTCCGTACTTCAGACTG | TsingKe |
| SMPD4 qPCR primer-R | TCGCTTTAGGAGGCTAGTGTG | TsingKe |
| β-ACTIN qPCR primer-F | CATGTACGTTGCTATCCAGGC | TsingKe |
| β-ACTIN qPCR primer-R | CTCCTTAATGTCACGCACGAT | TsingKe |
| MIC-A qPCR primer-F | CTTCAGAGTCATTGGCAGACAT | TsingKe |
| MIC-A qPCR primer-R | TGTGGTCACTCGTCCCAACT | TsingKe |
| MIC-B qPCR primer-F | TCTTCGTTACAACCTCATGGTG | TsingKe |
| MIC-B qPCR primer-R | TCCCAGGTCTTAGCTCCCAG | TsingKe |
| ULBP1 qPCR primer-F | TAAGTCCAGACCTGAACCACA | TsingKe |
| ULBP1 qPCR primer-R | TCCACCACGTCTCTTAGTGTT | TsingKe |
| ULBP2 qPCR primer-F | AGCAACTGCGTGACATTCAG | TsingKe |
| ULBP2 qPCR primer-R | GCCATCCTATACAGTCTCCCA | TsingKe |
| ULBP3 qPCR primer-F | TCTATGGGTCACCTAGAAGAGC | TsingKe |
| ULBP3 qPCR primer-R | TCCACTGGGTGTGAAATCCTC | TsingKe |
| PVR qPCR primer-F | TGGAGGTGACGCATGTGTC | TsingKe |
| PVR qPCR primer-R | GTTTGGACTCCGAATAGCTGG | TsingKe |
| BAG6 qPCR primer-F | AAGACCTTGGACTCTCAAACTCG | TsingKe |
| BAG6 qPCR primer-R | CCTGGTAAATGAGCCGTTGTTTT | TsingKe |
| CD48 qPCR primer-F | AGGTTGGGATTCGTGTCTGG | TsingKe |
| CD48 qPCR primer-R | AGTTGTTTGTAGTTCTCAGGCAG | TsingKe |
| ICAM1 qPCR primer-F | ATGCCCAGACATCTGTGTCC | TsingKe |
| ICAM1 qPCR primer-R | GGGGTCTCTATGCCCAACAA | TsingKe |
| NECTIN2 qPCR primer-F | GGATGTGCGAGTTCAAGTGCT | TsingKe |
| NECTIN2 qPCR primer-R | TGGGACCCATCTTAGGGTGG | TsingKe |
| NCR3LG1 qPCR primer-F | CTTTTATTCCCAACCCCTCAACA | TsingKe |
| NCR3LG1 qPCR primer-R | CACATCGGTACTCTCCTGCTT | TsingKe |
| IFNγ qPCR primer-F | TCGGTAACTGACTTGAATGTCCA | TsingKe |
| IFNγ qPCR primer-R | TCGCTTCCCTGTTTTAGCTGC | TsingKe |
| TNFα qPCR primer-F | CCTCTCTCTAATCAGCCCTCTG | TsingKe |
| TNFα qPCR primer-R | GAGGACCTGGGAGTAGATGAG | TsingKe |
| GZMB qPCR primer-F | CCCTGGGAAAACACTCACACA | TsingKe |
| GZMB qPCR primer-R | GCACAACTCAATGGTACTGTCG | TsingKe |
| PRF qPCR primer-F | GGCTGGACGTGACTCCTAAG | TsingKe |
| PRF qPCR primer-R | CTGGGTGGAGGCGTTGAAG | TsingKe |
| NKG2D qPCR primer-F | GAGTGATTTTTCAACACGATGGC | TsingKe |
| NKG2D qPCR primer-R | ACAGTAACTTTCGGTCAAGGGAA | TsingKe |
| NCR2 qPCR primer-F | GGCTCTCAGGCACAATCCAAG | TsingKe |
| NCR2 qPCR primer-R | GCTGAAGCCTCCTTACACCA | TsingKe |
| NCR3 qPCR primer-F | CCCCTGAGATTCGTACCCTG | TsingKe |
| NCR3 qPCR primer-R | CTCCACTCTGCACACGTAGAT | TsingKe |
| KLRF2 qPCR primer-F | TCCCAGAATGTAAACGTCAGC | TsingKe |
| KLRF2 qPCR primer-R | TTCCCTTCGTTCAACAGCCAG | TsingKe |
| GAPDH qPCR primer-F | CGACCACTTTGTCAAGCTCA | TsingKe |
| GAPDH qPCR primer-R | TTACTCCTTGGAGGCCATGT | TsingKe |
| pre-GAPDH qPCR primer-F | TGGGGACTGGCTTTCCCATAA | TsingKe |
| pre-GAPDH qPCR primer-R | GCCAGTAGAGGCAGGGATGA | TsingKe |
| sno-lncRNA qPCR primer-F | CTTGGCGTATTCATGGAGGT | TsingKe |
| sno-lncRNA qPCR primer-R | ACCGGCTAAGTGAGCTGAAA | TsingKe |
| LDHA qPCR primer-F | ATGGCAACTCTAAAGGATCAGC | TsingKe |
| LDHA qPCR primer-R | CCAACCCCAACAACTGTAATCT | TsingKe |

**Biological samples**

| Description | Source |
| --- | --- |
| Human HCC tumor tissue and paired liver tissue | Sir Run Run Shaw Hospital,  Zhejiang University |

**Reagents, kits, vectors etc.**

| Description | Source | Identifier |
| --- | --- | --- |
| WB/IP lysis buffer | Beyotime | Cat# P0013J |
| Anti-fluorescence quenching mounting solution | Beyotime | Cat# P0128M |
| Protein A+G Agarose | Beyotime | Cat# P2055 |
| TRIzol | TAKARA | Cat# 15596026 |
| Polybrene | Sigma-Aldrich | Cat# H9268 |
| Protease inhibitor cocktail | Abcam | Cat# ab65621 |
| Phosphatase inhibitor | Invitrogen | Cat# 78442 |
| Invitrogen™ ProLong™ gold antifade mountant with DAPI | Invitrogen | Cat# P36935 |
| Lipofectamine 3000 reagent | Invitrogen | Cat# L3000015 |
| Tyrpsin-EDTA (0.5%) | Invitrogen | Cat# 15400054 |
| Yeast tRNA (10 mg/mL) | Invitrogen | Cat# AM7119 |
| Streptavidin magnetic beads | Invitrogen | Cat# 88817 |
| RNase A | Invitrogen | Cat# EN0531 |
| Rnase R | Epicentral | Cat# RNR07250 |
| Ultrapure Salmon Sperm DNA | Invitrogen | Cat# 15632011 |
| RNase Inhibitor, recombinant (human placenta) | New England Biolabs | Cat# M0307S |
| Matrigel Basement Membrane Matrix | BD biosciences | Cat# 356237 |
| Actinomycin D | MedChemExpress | Cat# HY-17559 |
| Lactate | MedChemExpress | Cat# HY-B2227 |
| 2-Deoxy-D-glucose | MedChemExpress | Cat# HY-13966 |
| Cycloheximide | MedChemExpress | Cat# HY-12320 |
| Chloroquine | MedChemExpress | Cat# HY-17589A |
| Leupeptin hemisulfate | MedChemExpress | Cat# HY-18234A |
| TAK-243 | MedChemExpress | Cat# HY-100487 |
| PYR-41 | MedChemExpress | Cat# HY-13296 |
| MG-132 | MedChemExpress | Cat# HY-13259 |
| Nicotinamide | MedChemExpress | Cat# HY-B0150 |
| Trichostatin A | MedChemExpress | Cat# HY-15144 |
| Cell counting kit (CCK-8) | Yeasen Biotech | Cat# 40203ES60 |
| Endotoxin-free plasmid purification kit | Tiangen | Cat# DP117 |
| circRNA qRT-PCR kit | Geneseed | Cat# GS0201 |
| CellTrace CFSE Cell Proliferation Kit | Thermo Fisher | Cat# C34570 |
| PI/RNase Staining Buffer | BD Biosciences | Cat# 550825 |
| BDCytofix/Cytoperm™PlusFixation/Permeabilization Solution Kit with BD GolgiStop™ | BD Biosciences | Cat# 554715 |
| L-Lactic Acid (LA) Colorimetric Assay Kit | Elabscience | Cat# E-BC-K044-S |
| Pyruvic Acid Colorimetric Assay Kit | Elabscience | Cat# E-BC-K130-M |
| Fluorescent in situ hybridization kit | Ribobio | Cat# C10910 |
| RNA-binding protein immunoprecipitation kit | Millipore | Cat# 17-701 |
| Pierce™ Streptavidin Magnetic Beads | Invitrogen | Cat# 88817 |
| BCA protein assay kit | Invitrogen | Cat# M34152 |
| PARIS™ kit for nuclear and cytoplasmic RNA isolation | Invitrogen | Cat# AM1921 |
| Pierce™ Magnetic RNA-Protein pull-down kit | Invitrogen | Cat# 20164 |
| Competent cells: DH5α | Weidi Bio | N/A |
| Plasmid: pLO5-ciR | Geneseed | Cat# GS0107 |
| Plasmid: pLVX-Puro | Youbio | Cat# VT1465 |
| Plasmid: pLVX-shRNA2-Puro | Youbio | Cat# VT2240 |
| Plasmid: pcDNA3.1-3xFlag | Youbio | Cat# VT8001 |
| Plasmid: lentiCRISPRv2 | TsingKe | N/A |
| Plasmid: pLO5-ciR-circSMPD4 | This paper | N/A |
| Plasmid: pLVX-Puro-LDHA | This paper | N/A |
| Plasmid: pLVX-Puro-SMPD4 | This paper | N/A |
| Plasmid: pLVX-shLAMP2A-Puro | This paper | N/A |
| Plasmid: pcDNA3.1-SIRT2-3xFlag | This paper | N/A |
| Plasmid: pcDNA3.1-LDHA-3xFlag | This paper | N/A |
| Plasmid: pcDNA3.1-LDHA p.K5R-3xFlag | This paper | N/A |
| Plasmid: pcDNA3.1-LDHA p.K14R-3xFlag | This paper | N/A |
| Plasmid: pcDNA3.1-LDHA p.K57R-3xFlag | This paper | N/A |
| Plasmid: pcDNA3.1-LDHA p.K81R-3xFlag | This paper | N/A |
| Plasmid: pcDNA3.1-LDHA p.K118R-3xFlag | This paper | N/A |
| Plasmid: pcDNA3.1-LDHA p.K126R-3xFlag | This paper | N/A |
| Plasmid: pcDNA3.1-LDHA p.K222R-3xFlag | This paper | N/A |
| Plasmid: pcDNA3.1-LDHA p.K318R-3xFlag | This paper | N/A |
| Plasmid: pLO5-ciR-circSMPD4-LDHAmut  (67-72, 86-90, 396-398, 478-484, 496-509) | This paper | N/A |
| Plasmid: PCDH-Puro-LDHA-FL-3xFlag | This paper | N/A |
| Plasmid: PCDH-Puro-LDHA-ΔNTD-3xFlag | This paper | N/A |
| Plasmid: PCDH-Puro-LDHA-ΔCTD-3xFlag | This paper | N/A |
| Plasmid: PCDH-Puro-LDHB-FL-3xFlag | This paper | N/A |
| Plasmid: lentiCRISPRv2-circSMPD4 | This paper | N/A |
| Plasmid: lentiCRISPRv2-LDHA | This paper | N/A |
